# WEE1 kinase in cancer: Molecular mechanisms and inhibitor insights

**DOI:** 10.17179/excli2026-9568

**Published:** 2026-07-24

**Authors:** Ankush Kumar, Keshav Raj Paudel, Rajwinder Kaur, Rohit Bhatia

**Affiliations:** 1 Chitkara College of Pharmacy, Chitkara University, Rajpura-140401, Punjab, India; 2 NICM Health Research Institute and School of Science, Western Sydney University, NSW 2145, Australia

**Keywords:** WEE1 kinase, inhibitor, anticancer, cell cycle, checkpoint

## Abstract

WEE1 kinase is a main regulator of the G2/M cell cycle checkpoint. It plays an important role in maintaining genomic stability by inhibiting CDK1 through a phosphorylation process at Tyr15. WEE1 is found to be overexpressed in several cancers and also act as a protective mechanism that allows cancer cells to repair DNA damage and survive under replicative stress. So, pharmacological inhibition of WEE1 has emerged as a promising therapeutic strategy. Many conventional chemotherapeutic agents act by inducing DNA damage, so it enables the activation of WEE1 in cancer cells to arrest the cell cycle and repair this damage by preventing cell death. Inhibition of WEE1 disrupts this protective checkpoint, which ultimately leads to mitotic catastrophe. Therefore, targeting WEE1 represents a promising and rational therapeutic approach, mainly in tumors with TP53 mutations. We have comprehensively discussed the structural features of WEE1, its regulation in DNA damage response, epigenetic control, and its role in cancer progression. We have also summarized the clinical development of major WEE1 inhibitors such as adavosertib, azenosertib (ZN-c3), and Debio 0123. Moreover, recently synthesized small-molecule inhibitors are also discussed with special focus on structure-activity relationship (SAR) insights, dual-target inhibitors, and PROTACs and molecular glue-based degraders. Two compounds, 8 and 11, were found to be the most potent WEE1 inhibitors with excellent enzymatic inhibition. This explains the importance of rational scaffold optimization and electron-withdrawing group insertion for enhanced activity. Overall, this review serves as a valuable reference for medicinal chemists in the development of next-generation WEE1 inhibitors.

See also the graphical abstract(Fig. 1).

## Abbreviations

AML: Acute Myeloid Leukemia

ATM: Ataxia Telangiectasia Mutated

ATR: Ataxia Telangiectasia and Rad3-Related Protein

AUC: Area Under the Curve

CDC25: Cell Division Cycle 25 Phosphatase

CDK1: Cyclin-Dependent Kinase 1

CHK1: Checkpoint Kinase 1

CHK2: Checkpoint Kinase 2

CRBN: Cereblon

DDR: DNA Damage Response

GBM: Glioblastoma Multiforme

HDAC: Histone Deacetylase

HSP90: Heat Shock Protein 90

IC_50_: Half Maximal Inhibitory Concentration

Ki: Inhibition Constant

MTD: Maximum Tolerated Dose

NSCLC: Non-Small Cell Lung Cancer

PAMPA: Parallel Artificial Membrane Permeability Assay

PARP: Poly (ADP-Ribose) Polymerase

PDO: Patient-Derived Organoids

PEG: Polyethylene Glycol

PKMYT1: Protein Kinase Membrane Associated Tyrosine/Threonine 1

PROC: Platinum-Resistant Ovarian Cancer

PROTAC: Proteolysis Targeting Chimera

SAR: Structure-Activity Relationship

T1/2: Half-Life

TGI: Tumor Growth Inhibition

TP53: Tumor Protein p53

USC: Uterine Serous Carcinoma

VHL: Von Hippel-Lindau

WEE1: WEE1 G2 checkpoint kinase

## 1. Introduction

**Figure 2:**
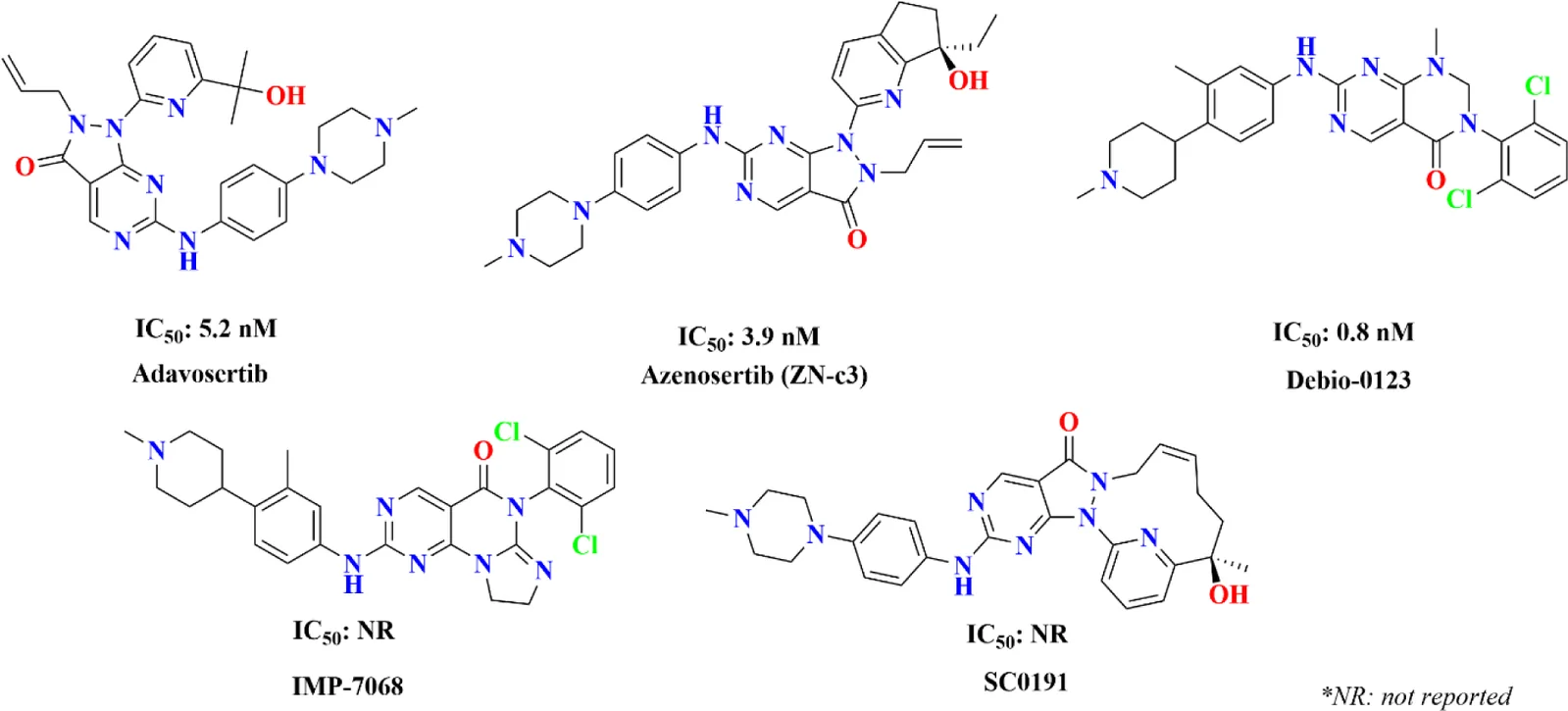
WEE1 kinase inhibitors in cancer treatment

Cancer still remains the second leading cause of death in the entire globe after cardiovascular-related diseases (ReFaey et al., 2021[53]; Vaduganathan et al., 2022[66]). In 2025, the United States is expected to see around 2 million new cancer diagnoses and over 618,000 deaths from cancer (Martin et al., 2025[38]). The emergence of targeted-based inhibitors has changed the field of cancer treatment (Jin et al., 2023[27]). Cancer is an uncontrolled division of cells due to an abnormal cell cycle (Matthews et al., 2022[40]). This abnormal cell cycle is caused by sudden mutations in pathway genes and arising defects in DNA repair pathways (Otto and Sicinski, 2017[49]; Pilié et al., 2019[52]). Checkpoint kinase 1 (CHK1), Checkpoint kinase 2 (CHK2), Ataxia telangiectasia and Rad3 related protein (ATR), Ataxia-Telangiectasia Mutated (ATM) and WEE1 are major cell cycle checkpoints (Tang et al., 2024[63]; Vlatkovic et al., 2022[69]). WEE1 is a serine/threonine kinase that plays an important role in the regulation of the G2/M cell cycle checkpoint (Koh, 2022[29]; Zhang et al., 2024[81]). This mechanism provides sufficient time for normal cells to repair DNA damage before mitotic progression (Zeng et al., 2022[79]). In cancer cells (with p53 deficiencies), the G2/M checkpoint becomes dependent on WEE1 activity for survival under replication stress (Melia and Parsons, 2024[42]; Pedroza‐Garcia et al., 2022[51]). Consequently, pharmacological inhibition of WEE1 forces damaged cancer cells into premature mitosis, leading to mitotic catastrophe and apoptotic cell death (Bukhari et al., 2022[5]). WEE1 negatively regulates cell cycle progression by phosphorylating CDK1, preventing a premature entry into mitosis (Koh, 2022[29]). WEE1 constitutes a major cell cycle checkpoint regulator. Targeting the WEE1 checkpoint has become a strong focus in anticancer drug development, and continuous efforts are being made by various researchers (Yan et al., 2022[77]; Zhang et al., 2025[83]). Presently, only a few WEE1 inhibitors are under clinical evaluation. However, they are demonstrating encouraging therapeutic efficacy. Moreover, when WEE1 inhibitors are combined with other therapies, they could lead to synergistic effects in some cancers with TP53 mutations (Liu et al., 2021[34]). Many WEE1 inhibitors were synthesized in the last decade and also showed good inhibitory activity. Figure 2(Fig. 2) displays the various WEE1 inhibitors used in cancer treatment.

## 2. Structural Representation of WEE1

**Figure 3:**
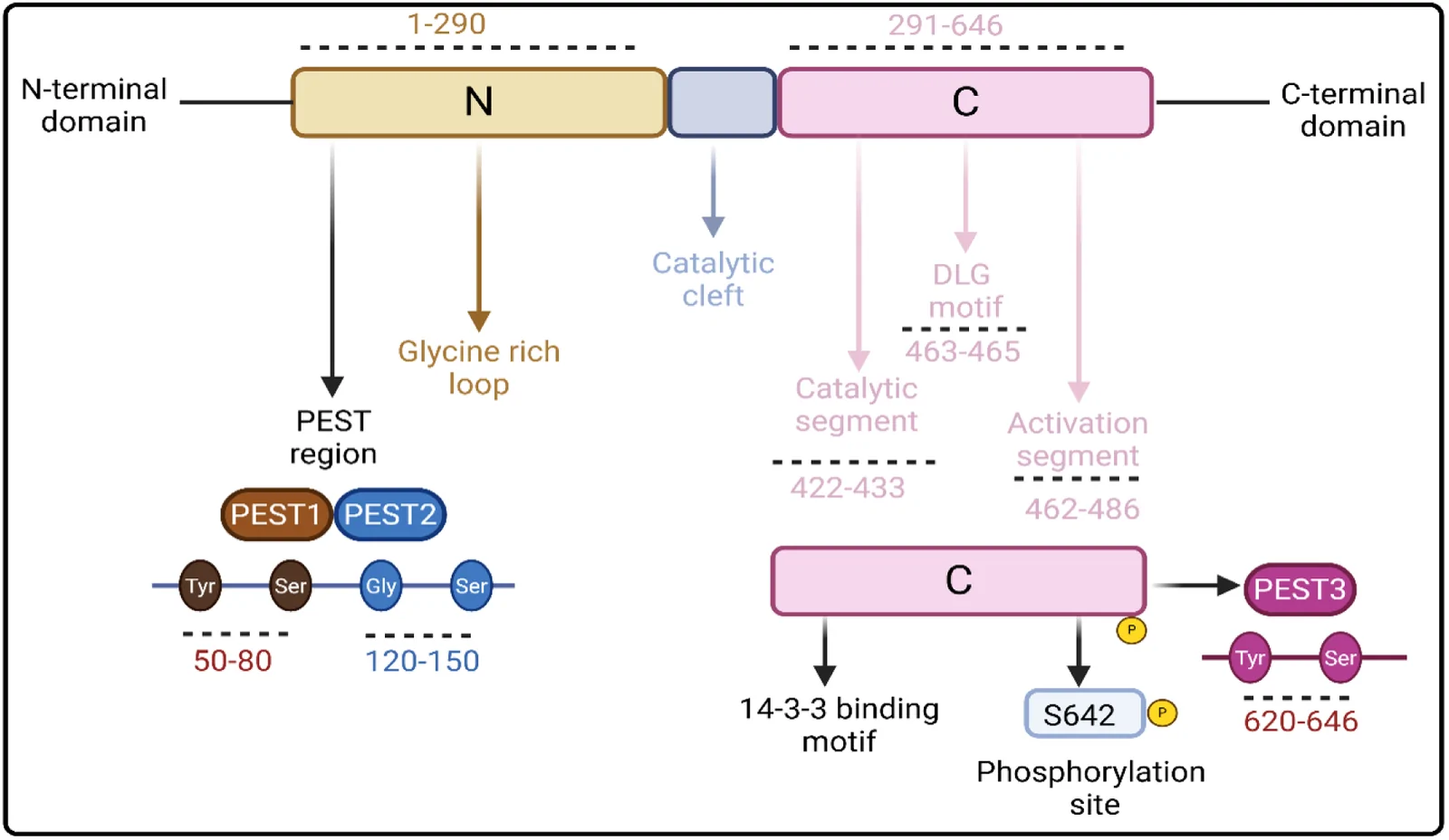
Structural features of WEE1 protein

The WEE1 gene was discovered by Paul Nurse in 1978 (Nurse and Thuriaux, 1980[48]). He identified this gene in fission yeast of *Schizosaccharomyces pombe* (Nurse and Thuriaux, 1980[48]). This discovery showed that the WEE1 gene is a negative regulator of mitosis and plays an important role in delaying the cell cycle. It is a nuclear serine/threonine specific type of kinase. WEE1 kinase consists of three main domains: N-terminal domain, catalytic cleft, and C-terminal domain, which contains 1-646 amino acid residues. The N-terminal lobe is composed of a glycine-rich loop containing 306-311 residues of amino acid. The C-terminal domain contains a catalytic segment having residues from 422 to 433, and one activation segment comprised of residues from 462 to 486 (Squire et al., 2005[60]). It also contains a DLG motif which provides a new approach for designing novel and selective WEE1 kinase inhibitors. Figure 3(Fig. 3) displays the structural representation of the WEE1 kinase. All the WEE1 domains contain many phosphorylation sites such as the P-site for PLK1 and CDK1. There is also one site for CK2 at S121 (Yde et al., 2008[78]). WEE1 kinase also contains three PEST regions (Watanabe et al., 1995[73]). PEST 1 and PEST 2 are present on the N-terminal domain with 50-80 and 120-150 amino acid sequences, respectively. PEST 3 is present on the C-terminal domain with 620-646 amino acid sequences. In addition to this, the C-terminal domain also contains an inhibitory P-site at S642 which promotes stability and nuclear export. Phosphorylation of S642 increases the binding of 14-3-3 and causes degradation or cytoplasmic retention (Guzman-Vendrell et al., 2015[21]; Watanabe et al., 2005[72]). This protein-binding motif binds chaperone proteins and prevents premature degradation (Goes and Martin, 2001[18]). The C-terminal domain is rich in serine and threonine, which act as phosphorylation sites (Owens et al., 2010[50]). All these structural features support or provide clues for medicinal chemists and researchers to synthesize small inhibitors for targeting WEE1 kinase in cancer therapy.

### 2.1 WEE1 regulation and impact on DNA damage repair and cell cycle

WEE1 is a serine/threonine type of kinase containing the WEE1 gene present on chromosome 11q14.3. It plays an important role in cell cycle regulation, mainly the transition of G2/M, by phosphorylating CDK1 at Tyr15. The cell cycle and its progression are regulated and controlled by CDKs. It acts as a key regulator of the G2/M checkpoint, acting by inhibiting the phosphorylation at Tyr15. It delays the CDK1-cyclin B activation and increases the mitotic entry timing until DNA is repaired. ichThat means, normal cells with incomplete replication and DNA damage do not initiate the cell cycle (Masuda et al., 2011[39]; Schmidt et al., 2017[56]). But in cancerous cells, WEE1 expression is upregulated by various oncogenes, and this upregulation seems like a compensatory response to DNA damage (Islam et al., 2023[26]). When DNA damage occurs, ATR activation phosphorylates the CHk1 on Ser345 and Ser317 amino acids (Ma et al., 2020[35]; Saldivar et al., 2017[55]). This phosphorylation activates and phosphorylates CDC25 and WEE1. Upon CDK1 phosphorylation on Tyr14 and Tyr15, WEE1 activation inhibits CDK1, causing G2/M arrest. This process stops the mitotic process till DNA damage is repaired (Saini et al., 2015[54]; Vera et al., 2015[67]).

**Figure 4:**
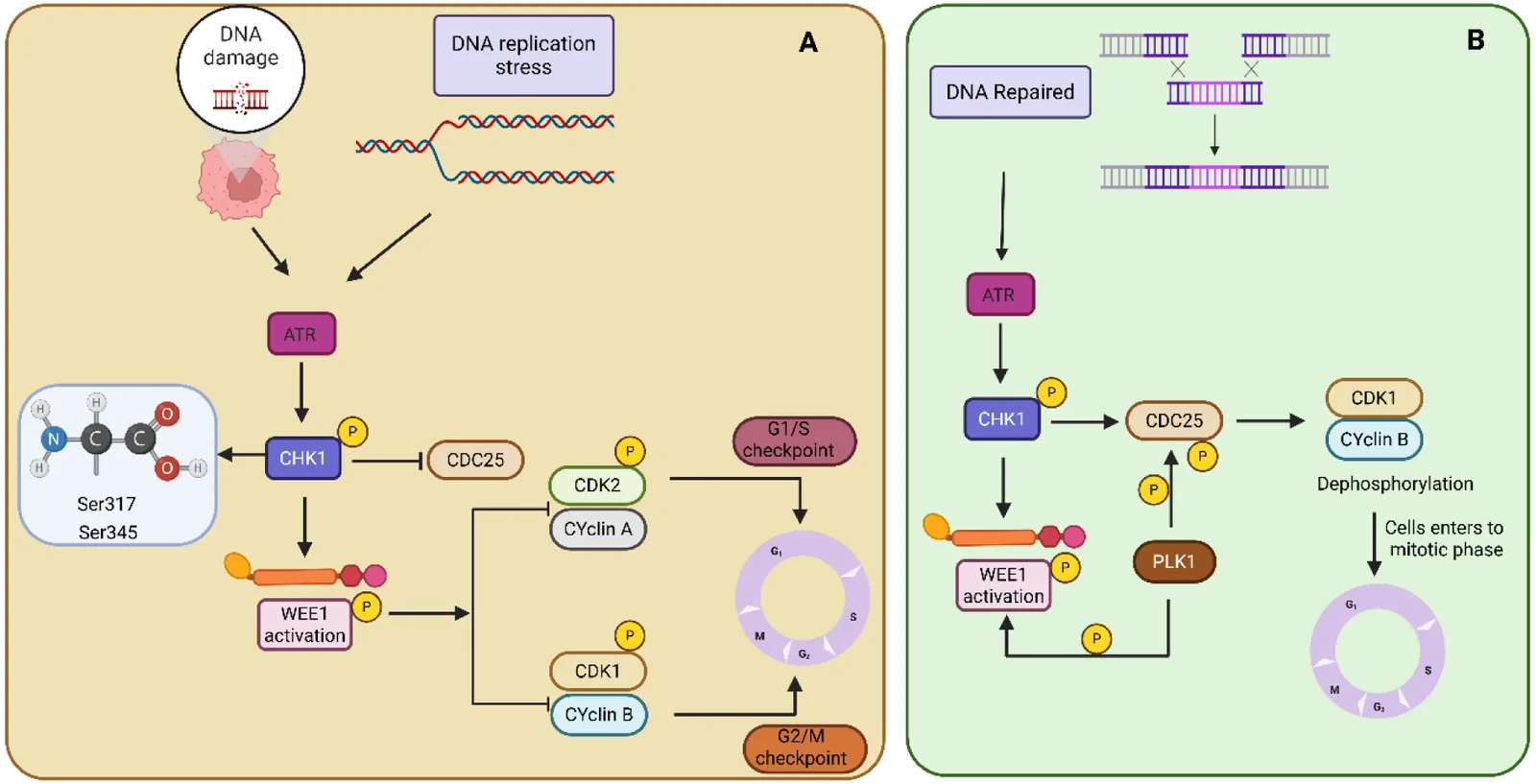
WEE1 regulation in DNA damage response and cell-cycle control: (A) DNA damage or replication stress activates the ATR-CHK1 pathway, leading to WEE1-mediated inhibition of CDKs and enforcement of cell-cycle checkpoints. (Brsid2169831 ) Following DNA repair, checkpoint signaling is relieved, resulting in CDK reactivation and progression into mitosis.

Once the DNA damage is repaired completely, CDK1 phosphorylation becomes weakened, and WEE1 activity decreases. Simultaneously, CHK1 reactivates CDC25 which dephosphorylates CDK1 and speeds up the entry of cells into the mitotic phase. Lastly, WEE1 and CDC25 act as key regulators that control the G2/M cell transition (Boutros et al., 2007[4]; Mueller and Haas-Kogan, 2015[46]). WEE1, upon activation, phosphorylates the CDK1 on Tyr15 during S phase. This phosphorylation prevents the S to G2 transition till DNA replication (Mahajan and Mahajan, 2013[37]). During S phase, WEE1 also phosphorylates H2B on Tyr37 and inhibits the histone genes. All these mentions suggest the WEE1 role as a major cell cycle regulator. Its inhibition causes abnormal mitosis, polypoid formation and cell death called “mitotic catastrophe” (Mahajan et al., 2012[36]; Mir et al., 2010[44]; Vitale et al., 2011[68]). Figure 4(Fig. 4) displays the WEE1 regulation in DNA damage response and cell-cycle control.

### 2.2 Functions of WEE1 in epigenetics and cancer

WEE1 kinase is a conserved type of nuclear tyrosine kinase that is activated during the S/G2 phase (Featherstone and Russell, 1991[14]; McGowan and Russell, 1995[41]). It plays an important role in epigenetic control in the regulation of histone gene expression. In both humans and mice, there are more than 50 genes coding for core histones in the Hist1 cluster. One of the major mechanisms involves the WEE1-dependent phosphorylation of histone H2B at tyrosine 37 (pY37-H2B). This prevents the binding of Nuclear Protein, Ataxia-telangiectasia locus (NPAT), which activates histone gene transcription. At the same time, this pY37-H2B modification results in the recruitment of a transcriptional repressor called “histone regulatory homology A” (HIRA). It is a part of the histone chaperone complex,05 which is the mammalian equivalent of yeast HIR proteins. Moreover, these proteins in yeast bind to the negative regulatory element (NEG) in histone genes and inhibit transcription. The pY37-H2B appears during the late S phase and disappears quickly once histone synthesis ends (due to the involvement of a phosphatase). All these observations present the dual role of WEE1 kinase as a surveyor of chromatin and mitotic gatekeeper as well (Mahajan and Mahajan, 2013[37]). WEE1 plays an important role in cell-cycle progression, histone synthesis, and genomic stability (Wuchty et al., 2011[76]). Gene expression studies have shown that WEE1 kinase is overexpressed in glioblastoma multiforme (GBM), making it an important target for cancer therapy (Iorns et al., 2009[25]). It is also found to be overexpressed in luminal and triple-negative breast cancers (Murrow et al., 2010[47]).

## 3. Clinical Development of WEE1 Inhibitors

WEE1 inhibitors are emerging as promising anticancer agents due to their ability to disrupt the G2/M cell-cycle checkpoint and selectively sensitize tumor cells with defective DNA damage response pathways. Among them, adavosertib (AZD1775) is the most extensively investigated compound and has progressed through multiple Phase I/II clinical trials in solid tumors and haematological malignancies (Cole et al., 2020[9]; Do et al., 2015[10]; Gatz et al., 2024[16]). Numerous Phase I/II trials have evaluated AZD1775 in combination with chemotherapeutic agents and radiotherapeutics. The results were found to enhance efficacy in ovarian cancer, uterine and neck cancers (Embaby et al., 2023[12]; Gatz et al., 2024[16]; Kong et al., 2020[30]; Liu et al., 2021[34]). These studies suggest that AZD1775 can be useful in overcoming treatment resistance by exploiting various processes such as TP53 mutations, replication stress, and defects in DNA repair. In a Phase I trial (NCT02037230, NCT02101775, NCT03968653), AZD1775 alone or combined with gemcitabine, cisplatin, or carboplatin defined tolerable dosing and achieved higher response rates in TP53-mutant tumors (21 %) compared with TP53 wild-type tumors (12 %). The most common side effects were fatigue, nausea, and hematologic toxicity. In a phase I, open-label, multicenter study, researchers evaluated the safety and optimal dosing of the WEE1 inhibitor adavosertib (AZD1775) combined with cisplatin. The trial aims to enhance DNA damage-induced tumor response while maintaining acceptable toxicity, with secondary assessment of disease-free survival and exploratory biomarker and quality-of-life outcomes. Primary outcomes are the recommended doses based on dose-limiting toxicity rates of 25 % within 42 days (Group A) and 30 % within 12 weeks (Group B). Secondary outcomes include disease-free survival in both groups, while exploratory endpoints assess pharmacodynamic effects, DNA damage biomarkers, surgical outcomes (Group A), and quality of life (Group B) (Kong et al., 2020[30]). A Phase II study in pediatric gliomas showed that AZD1775 increased the responses to radiation and chemotherapy with acceptable safety, which supports further evaluation of WEE1 inhibitors in pediatric cancers (Cole et al., 2023[8]). There are only four clinical trials to date that have evaluated the combination of WEE1 inhibitors with radiotherapy/radiation therapy. NCT03028766 evaluated AZD1775 with cisplatin-based chemoradiotherapy in head and neck cancer, focusing on safety and feasibility. In NCT04460937, adavosertib was combined directly with external beam radiotherapy as a radiation-focused sensitization strategy. NCT03345784 studied AZD1775 with cisplatin and radiotherapy in gynaecological cancers to enhance DNA damage during concurrent chemoradiation.

Newer, more selective WEE1 inhibitors such as azenozertib (ZN-c3), Debio-0123, and IMP7068 are currently under early-to-mid-stage clinical evaluation, aiming to improve therapeutic index and reduce toxicity. In a Phase I trial NCT04833582, azenosertib combined with gemcitabine displayed acceptable safety and promising activity in patients with relapsed/refractory osteosarcoma. The MTD dose was azenosertib 150 mg (5 days on/2 days off) with gemcitabine 800 mg/m^2^. It showed hematologic toxicity. The combination achieved 18-week event-free survival of 39 % and supports further Phase II evaluation (Avutu et al., 2024[2]). Preclinical studies showed that the combination of ZN-d5 (a Bcl-2 inhibitor) and azenosertib enhanced the inhibition of AML cells in TP53-mutant models. So, based on these findings, a Phase I/II open-label study (NCT05682170) was carried out to evaluate the safety and optimal dosing of azenosertib alone and in combination with ZN-d5 (Smith et al., 2023[59]). A clinical trial NCT05128825 was carried out on Zn-c3 in combination with platinum-resistant to determine the efficacy and safety in patients with ovarian or fallopian cancer. Zn-c3 (300-500 mg/day) showed a safety profile in 193 heavily pretreated patients with nausea, fatigue, and diarrhea as the most common TRAEs. Promising antitumor activity was observed in Cyclin E1-positive PROC and USC, which supports further clinical evaluation (Meric-Bernstam et al., 2025[43]). Additional next-generation candidates such as APR-1051 and other dual WEE1/PKMYT1 inhibitors have recently entered into first-in-human studies (Tolcher et al., 2025[64]). Additionally, this PHASE I/II study (NCT05765812) evaluated the WEE1 inhibitor Debio0123 in glioblastoma, with one arm incorporating temozolomide and concurrent radiotherapy (Gelderblom et al., 2023[17]). It showed early signals of tolerability in glioblastoma.

Overall, these clinical trials (displayed in Table 1(Tab. 1)) indicate that WEE1 inhibitors (AZD1775, Debio 0123, ZN-c3 etc.) exhibit a manageable safety profile. Toxicities are mainly gastrointestinal and hematologic. These agents significantly enhance the efficacy of chemotherapy and radiotherapy. The highest activity is observed in biomarker-enriched populations, mainly TP53-mutant and Cyclin E1-amplified tumors. Importantly, WEE1 inhibitors demonstrate promising radio-sensitizing potential. These findings support further biomarker-driven clinical development.

## 4. Recent Advancements in WEE1 Inhibitors

In recent years, significant progress has been made by various research groups in the development of novel inhibitors targeting WEE1 kinase. In this section, we have compiled and discussed recently synthesized small-molecule WEE1 inhibitors by highlighting their structural modifications, SAR trends, and biological evaluation. Compounds containing pyrazolopyrimidinones, pyrrolo-pyrimidines, tricyclic pyrimidines, and aminosulfonyl-substituted analogues, have displayed enhanced enzymatic potency with improved pharmacokinetic (PK) profiles. Additionally, we have also included recent studies in targeted protein degradation strategies, including PROTAC-based WEE1 degraders and molecular glue degraders. These approaches aim to enhance potency and selectivity and overcome resistance by promoting complete degradation of the WEE1 protein.

### 4.1 Recent advancements in WEE1 inhibitors

**Figure 5:**
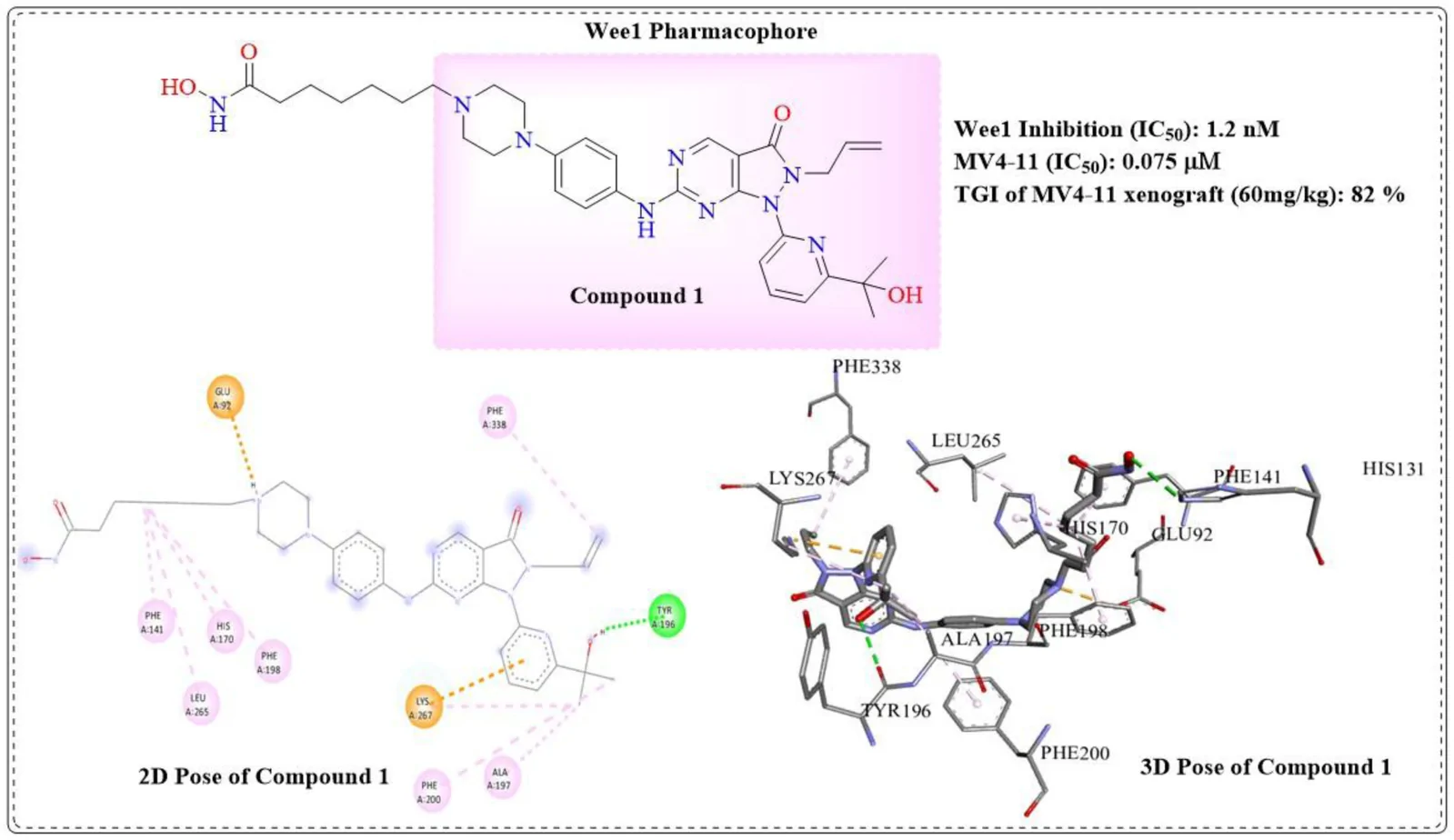
Chemical structure of compound 1 and 2D and 3D poses

Zhang and co-workers discovered a novel and potent dual WEE1/HDAC inhibitor for the treatment of AML (Zhang et al., 2025[82]). Additionally, it has been found that activation of CHK1 reduces the effectiveness of WEE1 inhibitors in the treatment of AML. To combat this, the authors have synthesized novel compounds with simultaneous inhibition of WEE1 and HDAC. Among all the synthesized compounds, compound 1 (as shown in Figure 5(Fig. 5)) displayed excellent WEE1 inhibitory potential. It displayed an IC_50_ of 1.2 nM. Additionally, compound 1 also inhibited MV4-11 with an inhibitory concentration of 0.075 µM. *In vivo* studies were also carried out in MV4-11 xenograft mice model. Compound 1 exhibited 82 % of TGI at a dose of 60 mg/kg. HDAC inhibitory potential was also carried out on three isoforms of HDAC. Compound 1 displayed HDAC1, HDAC3 and HDAC6 inhibitory potential of 0.196, 0.156 and 0.055 µM (Zhang et al., 2025[82]). Moreover, we have performed molecular docking studies on AutoDock software 1.4.6 to check the binding interactions of compound 1 with the WEE1 protein having a PDB ID of 5V5Y. The compound showed a binding score of -7.7 kcal/mol. 2D and 3D docking poses are displayed in Figure 5(Fig. 5). It can be observed that the terminal hydroxy group formed a hydrogen bond in interaction with Tyr196. Other hydrophobic interactions were seen with Phe141, His170, Phe198 and Phe338.

**Figure 6:**
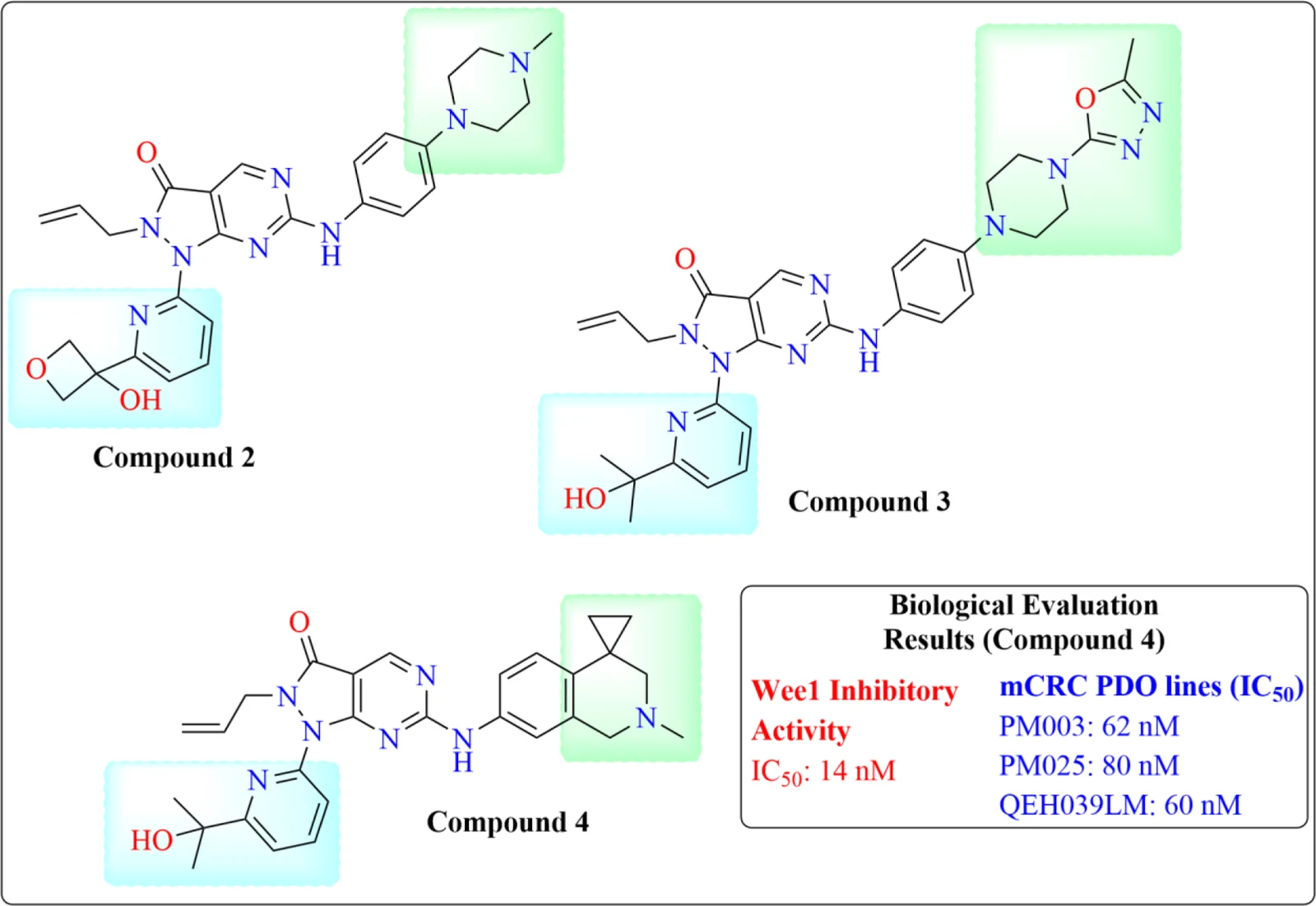
Chemical structures of WEE1 inhibitors designed by Syphers et al. (2025)

Syphers et al. discovered novel WEE1 inhibitors with potent inhibitory activity against metastatic colon cancer organoids (Syphers et al., 2025[61]). They have designed a library of compounds based on the previously developed candidate AZD1775. Biological activity was carried out on patient-derived organoids, and results showed that the three compounds 2, 3 and 4 showed remarkable inhibitory potential. Among them, compound 4 displayed excellent inhibitory activity with an IC_50_ value of 62 nM. Compound 4 also showed excellent WEE1 inhibitory potential and an IC_50 _value of 14 nM. This study provides the first PDO-based evidence showing that potent WEE1 inhibitors exhibit exceptional efficacy against CRC while enabling comparative assessment of their effects on malignant and healthy cells (Syphers et al., 2025[61]). Figure 6(Fig. 6) (Reference in Figure 6: Syphers et al., 2025[61]) displays the chemical structures of WEE1 inhibitors.

**Figure 7:**
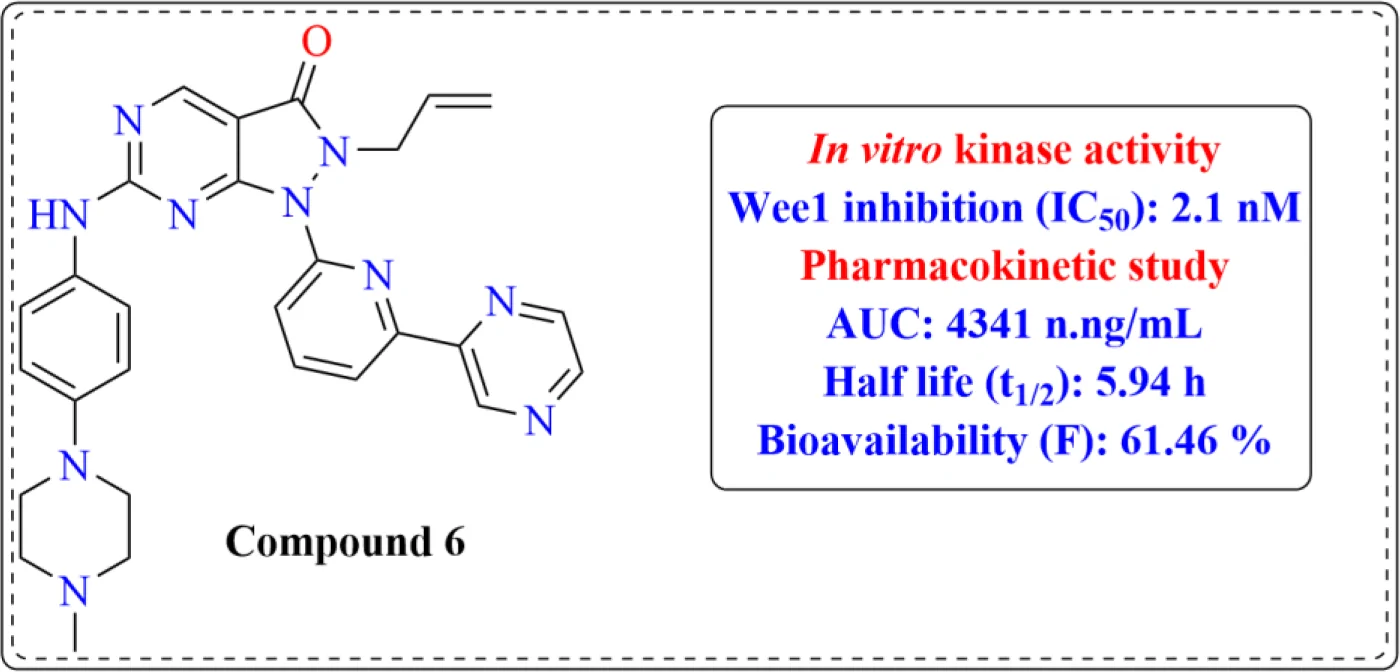
Chemical structure of selective WEE1 inhibitors synthesized by Wang et al. (2024b)

Previous studies have displayed AZD1775 as having poor kinase activity and dose-limiting toxicity. To get rid of this, Wang and co-workers synthesized highly selective WEE1 inhibitors (displayed in Figure 7(Fig. 7); Reference in Figure 7: Wang et al., 2024[71]). Among the synthesized derivatives, compound 5 displayed potent Wee1 inhibitory activity with an IC_50_ of 2.1 nM. *In vivo* PK studies were carried out in mice through intragastric administration of compound 6. Results displayed an AUC of 4342 h/ng/ml and a half-life (*t*_1/2_) of 5.94 h. It also displayed bioavailability of 61.46 %. These results suggest that compound 6 could be used as a promising drug candidate for the treatment of cancer (Wang et al., 2024[71]).

**Figure 8:**
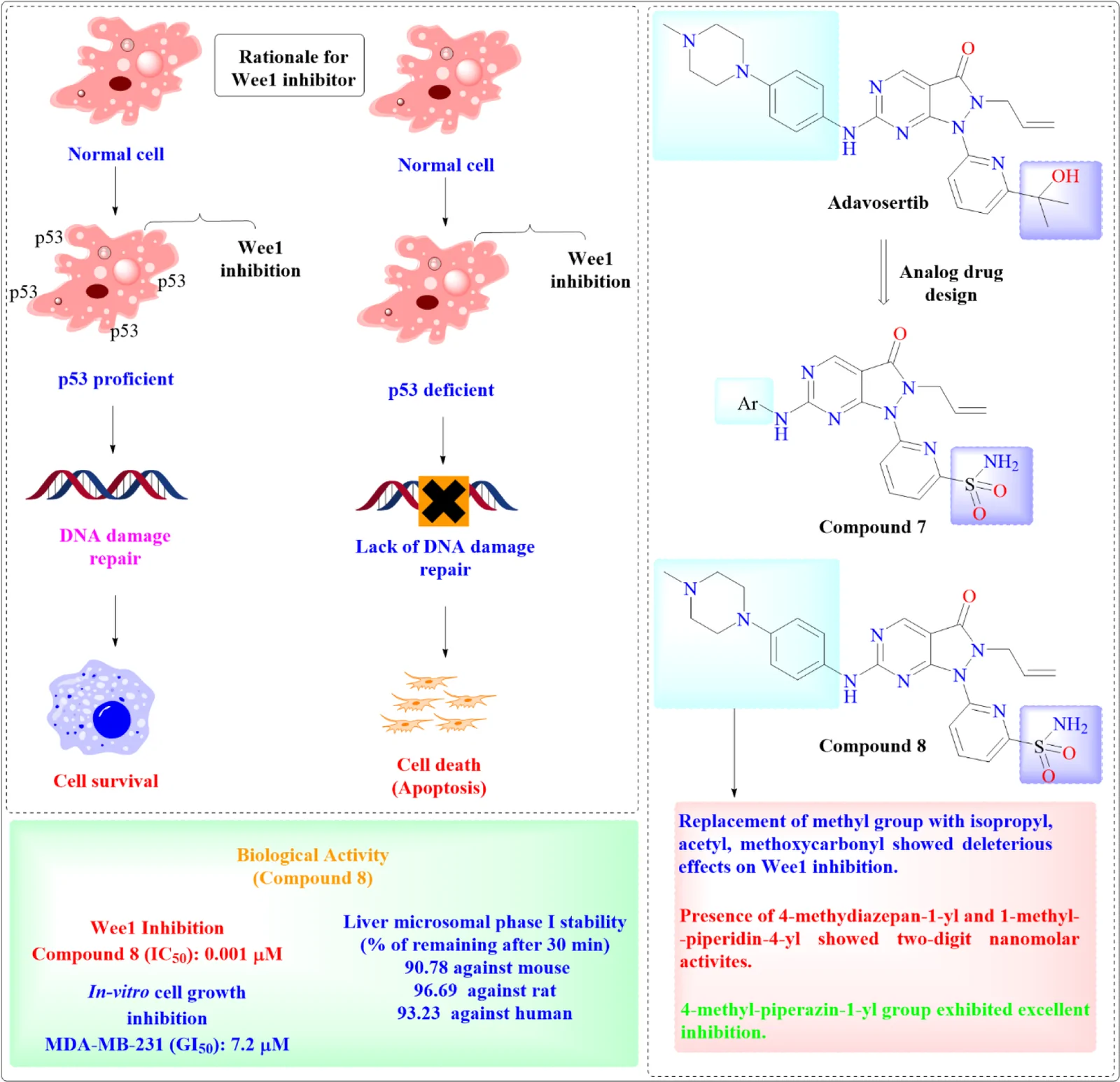
Rationale design and chemical structure of WEE1 inhibitors synthesized by Kim et al. (2024)

Many WEE1 inhibitors have been identified over the past fifteen years, and only four compounds have entered clinical trials (Du et al., 2020[11]). Among them, a compound known as adavosertib is developed by AstraZeneca, under phase III for the treatment of Pt-resistant TP53- cancer (Hirai et al., 2009[22]). However, in 2020, AstraZeneca discontinued adavosertib owing to strategic considerations (Liu et al., 2021[34]). Zentalis Pharmaceuticals has developed azenosrtin (currently in Phase II trial) for the treatment of advanced or metastatic solid tumors, and also in combination with gemcitabine (I/II) for the treatment of osteosarcoma in adult and pediatric patients (Huang et al., 2021[23]). Based on the various properties of adavosertib, Kim and co-workers synthesized 2-aminosulfonylpyridin-6-yl)pyrazolopyrimidinone derivatives through analog drug design as potent WEE1 inhibitors for the treatment of cancer (Kim et al., 2024[28]). The authors have designed molecules containing an aminosulfonyl group instead of the 2-hydroxypropan-2-yl moiety, which is already present in adavosertib. The similarity between the groups is the pseudotetrahedral configuration and the N-H hydrogen bond donor. Among all the synthesized derivatives (7), compound 8 was found to be potent. It displayed an excellent WEE1 inhibitory potential of 0.0001 µM. The solubility and permeability of compound 8 were evaluated to understand its MDA-MB-231 cell growth inhibitory activity and WEE1 substrate phosphorylation inhibition. The reduced cellular activity of compound 8, compared with adavosertib, may be attributed to its lower permeability (PAMPA (logP_e_): -5.890 vs 4.168, respectively). Also, the semi-equilibrium solubility of compound 8 was found to be 117.1 and 174.6 µSOL, µM for adavosertib (Kim et al., 2024[28]). Rational design and chemical structures of the potent compound are displayed in Figure 8(Fig. 8) (Reference in Figure 8: Kim et al., 2024[28]).

**Figure 9:**
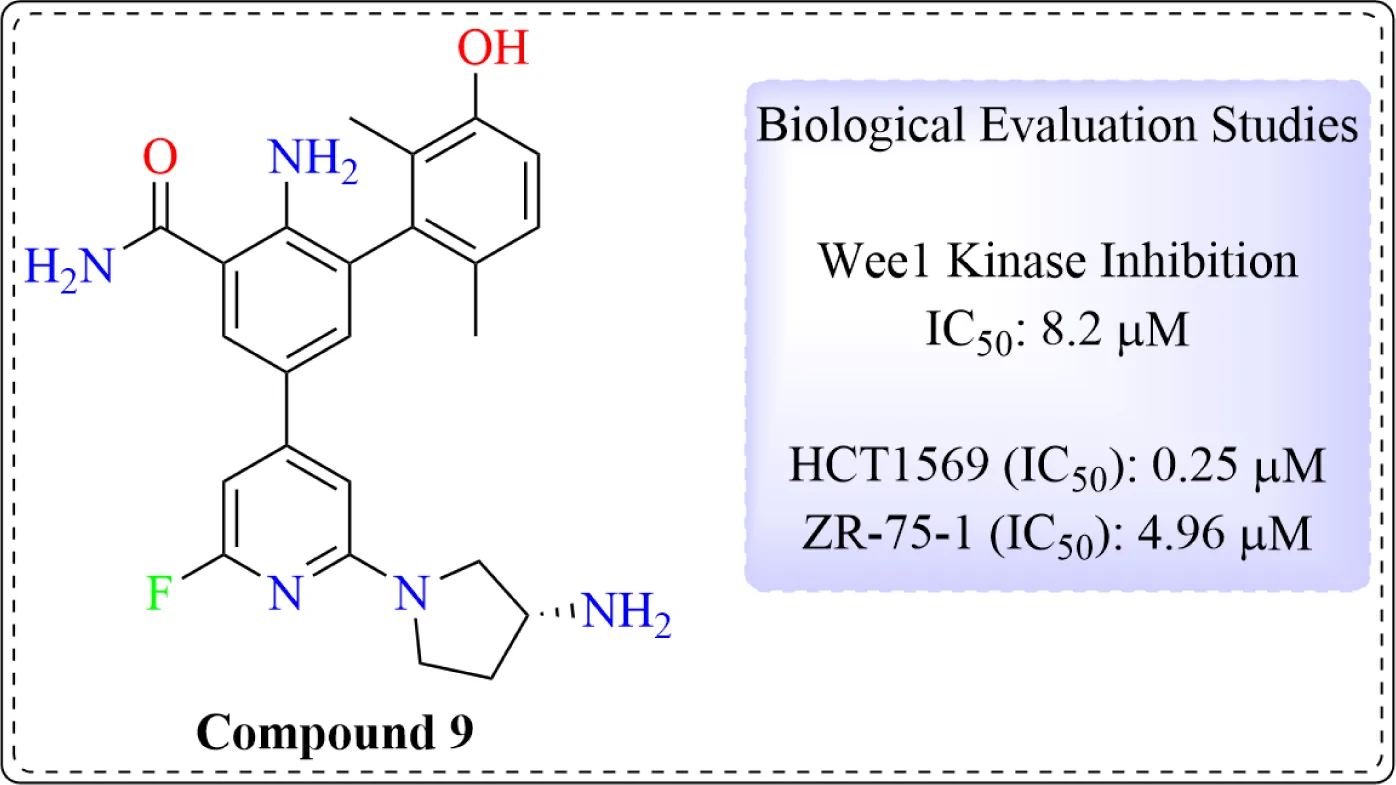
Chemical structure of 2-Amino-[1,1′-biphenyl]-3-carboxamide derivative

Wang et al. synthesized 2-Amino-[1,1′-biphenyl]-3-carboxamide derivatives for the treatment of breast cancer. WEE1 inhibitory activity was carried out and compound 9 displayed an IC_50_ value of 8.2 µM. Anticancer activity was carried out on CCNE1-amplified HCC1569 cell lines, and results displayed an IC_50_ of 0.25 µM (Wang et al., 2024[70]). Figure 9(Fig. 9) displays the chemical structure of compound 9.

**Figure 10:**
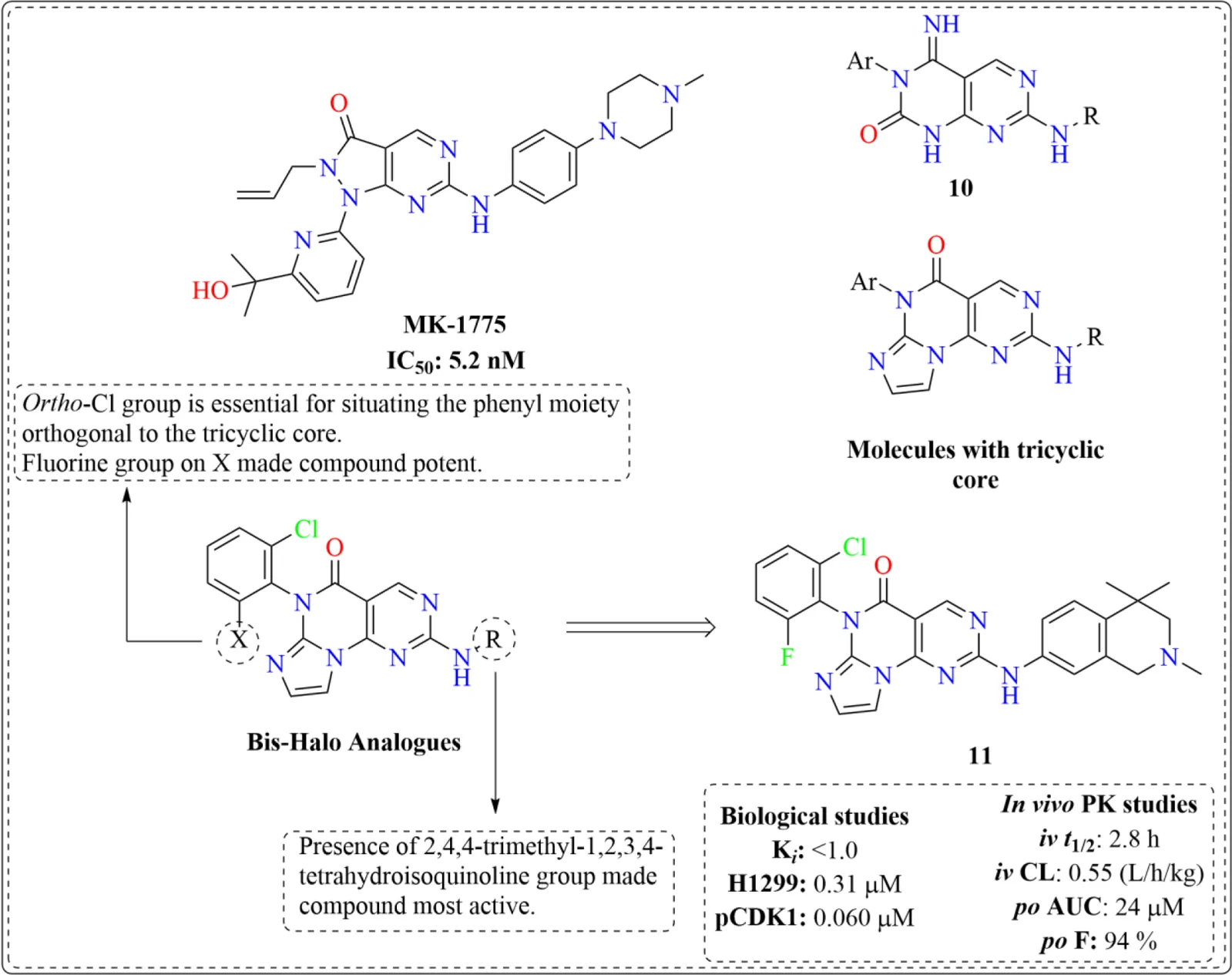
Chemical structures and SAR of WEE1 inhibitors

MK-1775 is a selective WEE1 inhibitor containing a 1*H*-pyrazolo[3,4-*d*] pyrimidin-3(2*H*)-one scaffold (Mizuarai et al., 2009[45]). It displayed antitumor activity with an IC_50_ of 5.2 nM (Tong et al., 2015[65]). Imino-dihydropyrimidinone pyrimidine cores containing WEE1 inhibitors are also available in the literature (Tong et al., 2015[65]). Computer-aided drug design revealed a key region (not utilized by the previously synthesized compound 10) within the ATP pocket in the WEE1 protein. This region is present adjacent to the bicyclic core of MK-1775 and is occupied by the pyridyl moiety. By taking advantage of this region, the authors have carried out a *de novo* drug design and molecular modeling approach to identify new WEE1 inhibitors. They have synthesized two series named mono-Cl and bis-halo analogues. Mono-Cl analogues displayed WEE1 *K*_i_ values in the range of 1.2-3.14 nM. Another Bis-Halo analogue series displayed *K*_i_ values in the range of 1.0-2.0 nM. Among all the synthesized compounds, compound 11 displayed excellent WEE1 inhibitory activity with a *K*_i_ value of less than 1.0 nM. Compound 11 also possessed a half-life of 2.8 h, low clearance of 0.55 L/h/kg, and excellent oral bioavailability of 94 %. Further, the pY15 Cdk1.2 marker of WEE1 inhibition was also determined by orally dosing (100 mg/kg/day) of compound 11. Results showed that it caused complete depletion of the pY15 band after 6 and 10 h of dosing. *In vivo* cytotoxic activity was evaluated against the H1299 mouse xenograft model in combination with irinotecan. Results displayed that the combination of irinotecan and compound 11 showed a TGI of 83 %. All these results displayed that the tricyclic core may have inhibitory potential for the treatment of cancer (Tong et al., 2015[65]). Chemical structure and SAR are displayed in Figure 10(Fig. 10).

**Figure 11:**
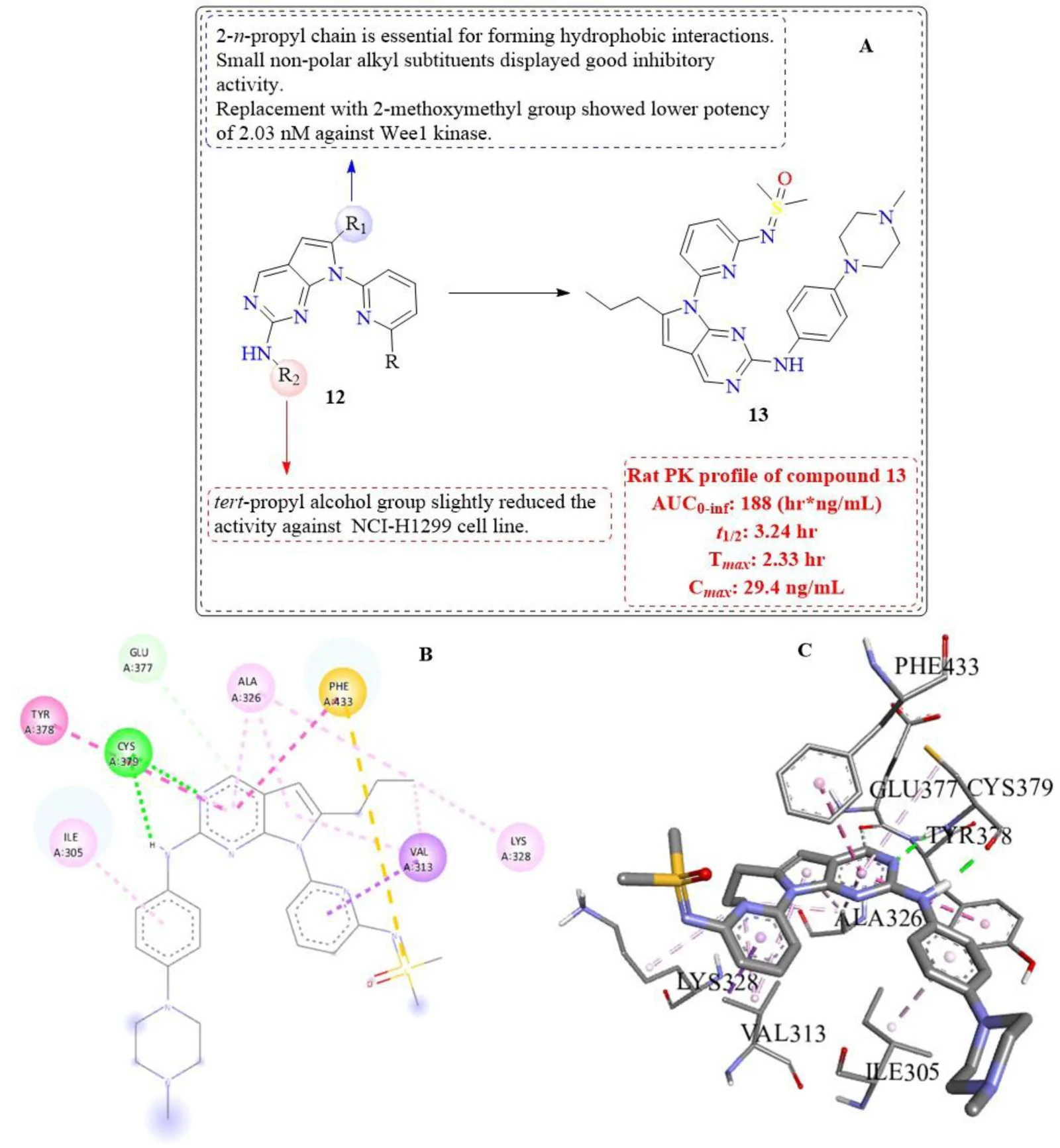
Chemical structures of compound 12 and 13 (insrsid16261485A); (B) and (C) display the 2D and 3D poses of compound 13.

Several WEE1 inhibitors such as AZD1775 and Zn-c3 contain a pyrazolopyrimidinone core. The pyrazolopyrimidinone core of AZD1775 forms two hydrogen bond interactions with Cys379 and Asn376 amino acids. Other hydrophobic interactions were also formed with Ile305, Val313 and Phe443 (Zhu et al., 2017[86]). Inspired by previously synthesized pyrazolopyrimidinones, Chen et al., synthesized pyrrolo[2,3-*d*]pyrimidine derivatives (11) as potent WEE1 inhibitors. Among all the synthesized compounds, compound 13 displayed excellent WEE1 inhibitory activity with an IC_50_ of 0.76. *In vitro* anticancer activity was also performed on NCI-H1299, and compound 13 displayed an IC_50_ of 33.1 nM, which is comparable to the reference compound AZS1775 (104 nM). SAR studies (shown in Figure 11A(Fig. 11)) displayed that 2-*n*-propyl group at R_1_ made the compound active (Chen et al., 2022[7]). To determine the binding interactions of the compound with the protein, we performed docking studies on the WEE1 protein (PDB ID: 7N3U). Compound 13 showed a binding score of -9.0 kcal/mol. 2D (B) and 3D (C) poses are displayed in Figure 11(Fig. 11). The 2-*n*-propyl group formed hydrophobic interactions with Val313. The pyrrolo[2,3-*d*]pyrimidine ring formed hydrophobic interactions with Ala326. The NH of the amine linker formed a hydrogen bond interaction with the Cys379 amino acid. Overall, compound 13 emerges as a promising WEE1 inhibitor. It showed potent enzymatic and cellular activity supported by SAR and molecular docking studies. The key interactions observed within the WEE1 binding pocket rationalize its enhanced potency and provide a basis for further scaffold optimization.

### 4.2 Protein degraders-based approaches for WEE1 kinase

Targeted protein degradation is a powerful approach for targeting specific proteins to treat diseases (Zhao et al., 2022[84]). It acts through two processes, such as ubiquitination and proteasome-mediated degradation. In medicinal chemistry, mainly two types of strategies are being followed (Zhong et al., 2024[85]). PROTACs are bifunctional molecules that are composed of a target-protein recruiting moiety and an E3 ligase moiety through a flexible linker. One part binds to the protein and another part binds to the E3 ubiquitin ligase and helps to bring them together in close proximity (Wells and Kumru, 2024[74]). After attaching, the E3 ligase starts sending signals for the destruction of the protein, and the ubiquitinated target protein is broken down by the cell's proteasome. PROTACs mainly eliminate the protein completely and are also helpful in overcoming resistance. On the other hand, molecular glues are small monovalent molecules that interact between the target protein and the E3 ubiquitin ligase. They also lead to protein ubiquitination and degradation in a proteasome-dependent manner (Bond and Crews, 2021[3]; Hughes and Ciulli, 2017[24]; Schreiber, 2021[57]).

#### 4.2.1 PROTAC-based approaches for WEE1 kinase

**Figure 12:**
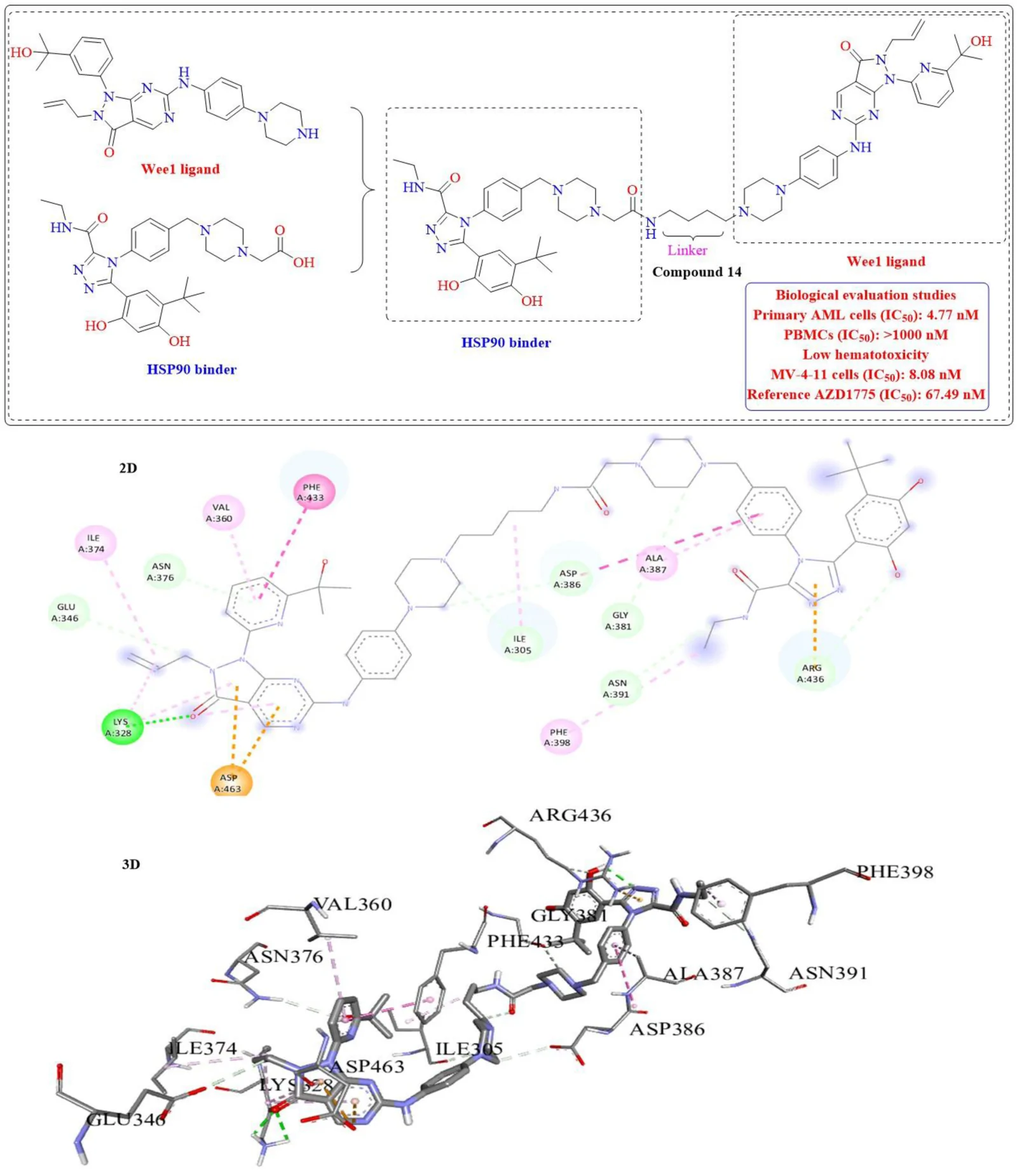
Chemical structure and SAR of Compound 14; 2D and 3D docking poses of the compound with WEE1 protein (PDB ID: 8BJU)

AZD1775 (WEE1 ligand) represents a promising target-binding ligand for the development of PROTACs aimed at selectively inducing WEE1 degradation. Li and co-workers synthesized WEE1 degraders via Hsp90-mediated targeting chimeras for the treatment of AML (Li et al., 2025[32]). Among the synthesized degraders, compound 14 effectively degraded the cellular WEE1 protein. Anticancer activity was carried out on AML cells and displayed an IC_50_ of 4.77 nM. Compound 14 also showed low hematotoxicity. It also displayed MV-4-11 inhibitory activity of 8.08 nM. It also induced cell cycle arrest in the G2/M phase in the MV-4-11 cells. Also, compound 14 (3 mg/kg) displayed excellent *in vivo* anticancer activity in AML patient-derived xenografts in mice. The chemical structure and design strategy of compound 14 are displayed in Figure 12(Fig. 12). To determine the binding interactions of compound 14 with the WEE1 protein, we performed docking studies on the WEE1 protein with PDB ID of 8BJU, and found a docking score of -8.9 kcal/mol. 2D and 3D poses are displayed in Figure 12(Fig. 12). Docking results showed that the carbonyl group formed a hydrogen bond interaction with Lys328. Other hydrophobic interactions were seen with Val360, Ile374 and Phe433. 4-carbon linker formed hydrophobic interactions with Ile305 amino acid. Overall, the combined *in vitro*, *in vivo* and *in silico* docking studies confirm that compound 5 is a potent and selective WEE1 degrader with strong anti-AML activity. These findings display its potential as a promising lead candidate for further preclinical development.

**Figure 13:**
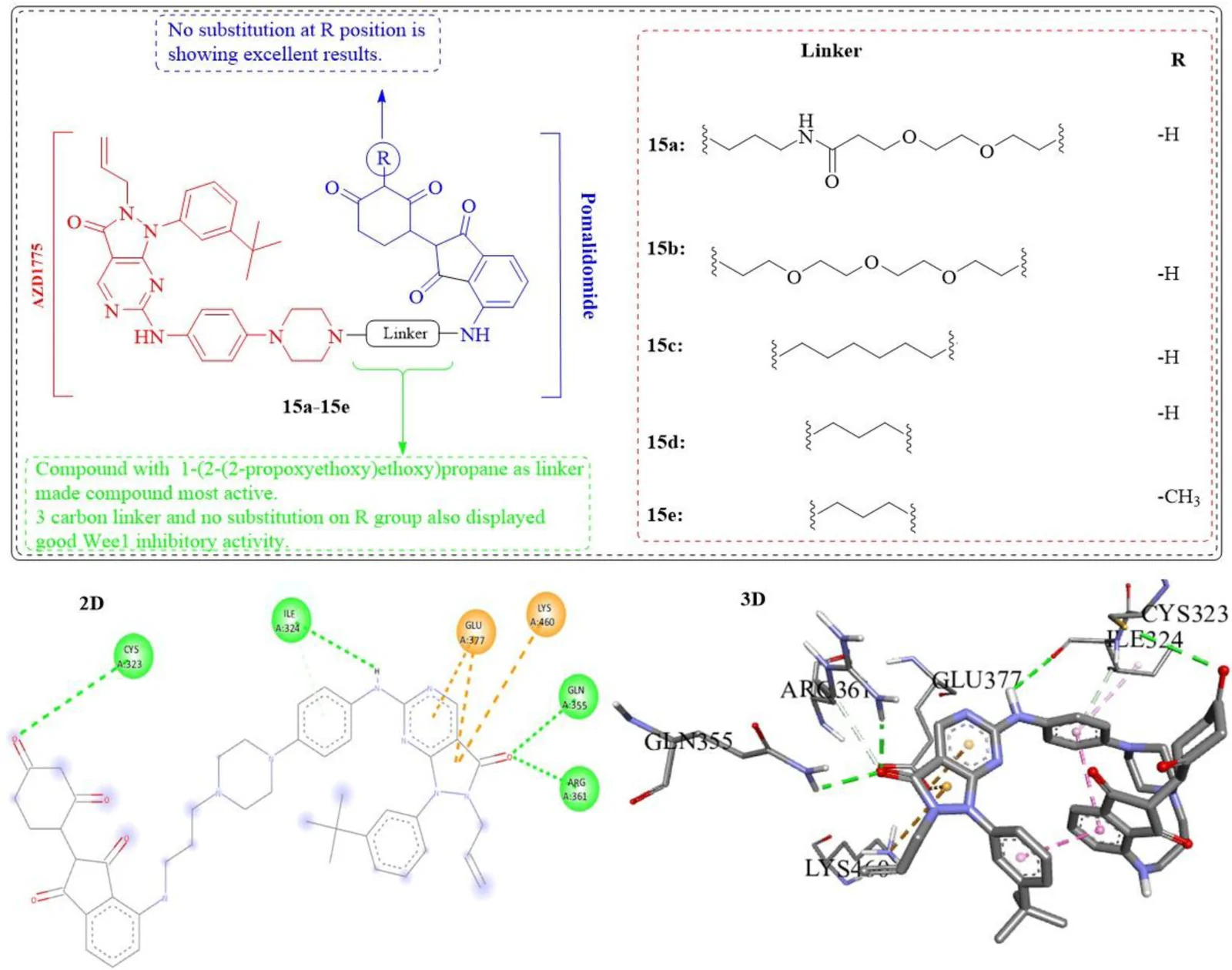
Chemical structure and SAR of Compound 15a-e; 2D and 3D docking poses of the compound with WEE1 protein (PDB ID: 8BJU)

Li Zhengnian and co-workers developed WEE1 kinase degraders by combining AZD1775 and pomalidomide through a linker. AZD1775 is a WEE1 inhibitor which decreases the CDK1 Tyr15 phosphorylation (Li et al., 2020[33]). AZD1775 induces mitosis and causes tumor regression in preclinical cancer models (Fu et al., 2018[15]). AZD1775 displayed good inhibitory results, but it is also associated with many dose-limiting toxicities such as thrombocytopenia, anemia and neutropenia (Do et al., 2015[10]; Guertin et al., 2013[19]). Bifunctional degraders are composed of an E3 ligase and another protein-binding ligand, which bind to the protein of interest. They offer more advantages compared to the traditional inhibitors, because they have the ability to selectively degrade the protein. In the work by Li et al., the authors have developed degraders by conjugating AZD1775 to pomalidomide. The *N*-methylpiperazine of AZD1775 is found to be solvent-exposed, so the authors have selected this site for attachment of the pomalidomide linker to the AZD1775. For this, the authors have synthesized various derivatives (15a-15e) with 2-6 carbon chain linkers. Among all the synthesized compounds, compound 15d with a three-carbon linker induced excellent WEE1 degradation with a 10-fold lower dose as compared to the inhibitor. It also displayed an excellent IC_50_ value of 390 nM against MOLT4 cells as compared to AZD1775 (800 nM). Also, the authors have checked the antiproliferative activity of compound 15d on a pool of 300 cell lines. It displayed submicromolar values in all the cells (Li et al., 2020[33]). Docking studies were carried out on the 8BJU protein. Figure 13(Fig. 13) displays the chemical structure and SAR of compounds 15a-e.

**Figure 14:**
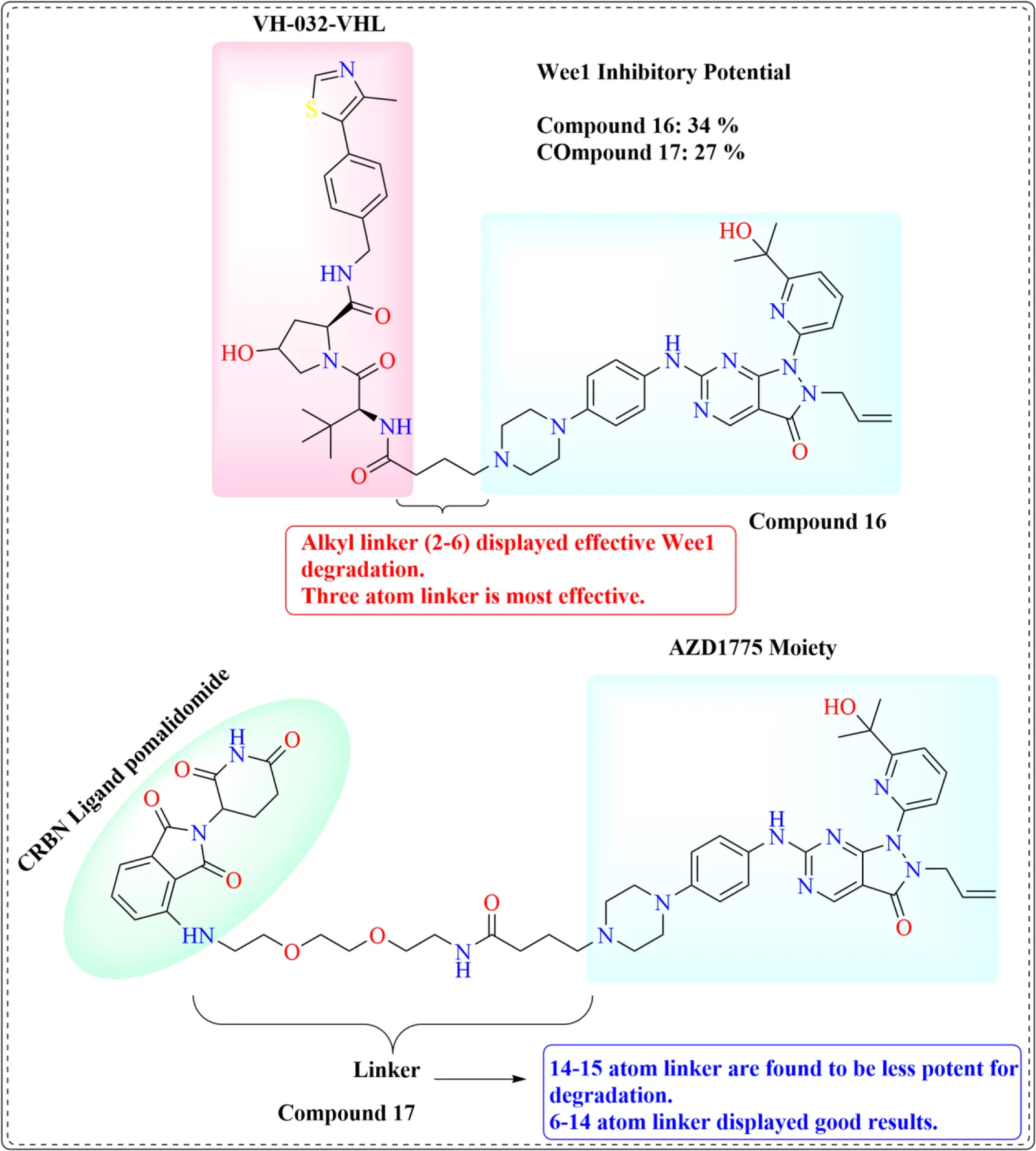
Chemical structures of WEE1-PROTACs

Aublette and co-workers described the synthesis of a series of WEE1-PROTACs using AZD1775 linked to either a CRBN ligand (pomalidomide) or a VHL ligand (VH032). The authors synthesized various derivatives by introducing different types and lengths of linkers for SAR optimization. Mainly, modifications were done at the 4-methylpiperazine region of AZD1775. SAR studies showed that linker length and type of linker critically influence the activity. In the VHL series, a compound with a three-atom linker (compound 16) displayed a strong degradation of approximately 34 %, while intermediate linkers ranging from 8-12 atoms were found to be less effective. Compounds with PEG linkers made the compounds inactive. In the CRBN series (compound 17), short to moderately short linkers with 13-15 atoms showed less degradation activity, while PEG linker-containing compounds with additional amide bonds were found to be poor protein degraders. Linkers with 6-14 atoms displayed excellent degradation. Overall, the study showed that short alkyl linkers are more favorable then medium-length linkers. PEG linkers diminish the activity and show less degradation. It suggests that in WEE1-PROTACs, linker architecture plays a critical role in efficient degradation (Aublette et al., 2022[1]). Chemical structures are shown in Figure 14(Fig. 14).

#### 4.2.2 Molecular glue degrader as WEE1 inhibitor

**Figure 15:**
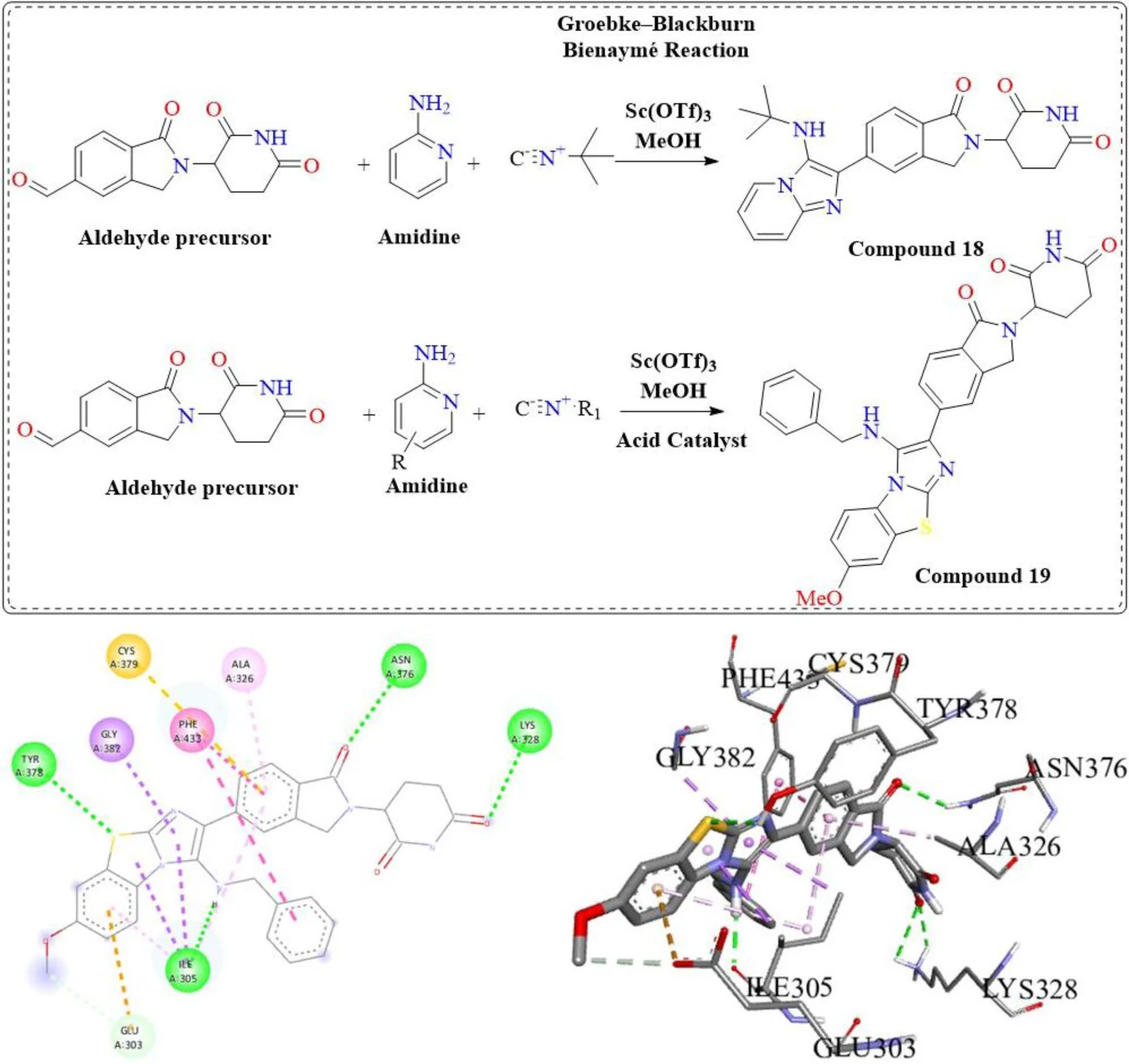
Synthetic process of molecular glue degraders and docking poses of compound 19

Molecular glue degraders are small molecules that induce the interaction between the target protein and the E3 ubiquitin ligase. This leads to ubiquitination and subsequent proteasomal degradation of the target protein. As compared to PROTACs, glue degraders are monovalent small molecules that stabilize the new protein-protein interaction between the target protein WEE1 and the E3 ligase. This results in selective degradation of the WEE1 target via the ubiquitin-proteasome system. Hlib et al. synthesized molecular glue degraders as potent WEE1 inhibitors. Two compounds, 18 and 19, displayed excellent antiproliferative activity of 12 and 139 nM (chemical structures are shown in Figure 15(Fig. 15)). They synthesized compounds 1 and 2 by reacting 2-(2,6-dioxopiperidin-3-yl)-1-oxoisoindoline-5-carbaldehyde with pyridine-amine in the presence of substituted cyanides to form CRBN-dependent WEE1 degraders. Compound 18 also causes degradation of both WEE1 and CK1alpha. SAR studies showed that the linkage of the aromatic system is responsible for WEE1 selectivity. The presence of hydrophobic substitutions at the amine group affects the potency and selectivity of WEE1 degradation. Molecular docking studies of compound 19 were performed on the 8BJU protein with a docking score of -10.9 kcal/mol. Docking studies revealed that the terminal carbonyl group formed an H-bond interaction with the Lys328 amino acid. The methoxy group also formed a hydrogen bond with Glu303.

## 5. Potential Resistance Mechanisms to WEE1 Inhibitors

Cancer cells can evade the cytotoxic effects of WEE1 inhibition through activation of compensatory signaling pathways, alterations in cell-cycle regulation, and adaptive rewiring of DNA damage responses. One well-characterized mechanism involves the upregulation of the WEE1-related kinase MYT1, which functionally compensates for WEE1 by maintaining inhibitory phosphorylation of CDK1, thereby preserving G2/M checkpoint control despite pharmacological inhibition (Lewis et al., 2019[31]). Additionally, activation of receptor tyrosine kinase signaling, particularly via AXL, has been shown to sustain downstream mTOR and ERK signaling, which in turn promotes CHK1 activation and reinforces parallel checkpoint pathways that bypass WEE1 dependency (Sen et al., 2017[58]). Moreover, WEE1 inhibition induces replication stress and consequent activation of the ATR-CHK1 pathway, which acts as a critical survival axis by stabilizing replication forks and limiting DNA damage, thereby reducing drug sensitivity (Bukhari et al., 2019[6]).

**Figure 16:**
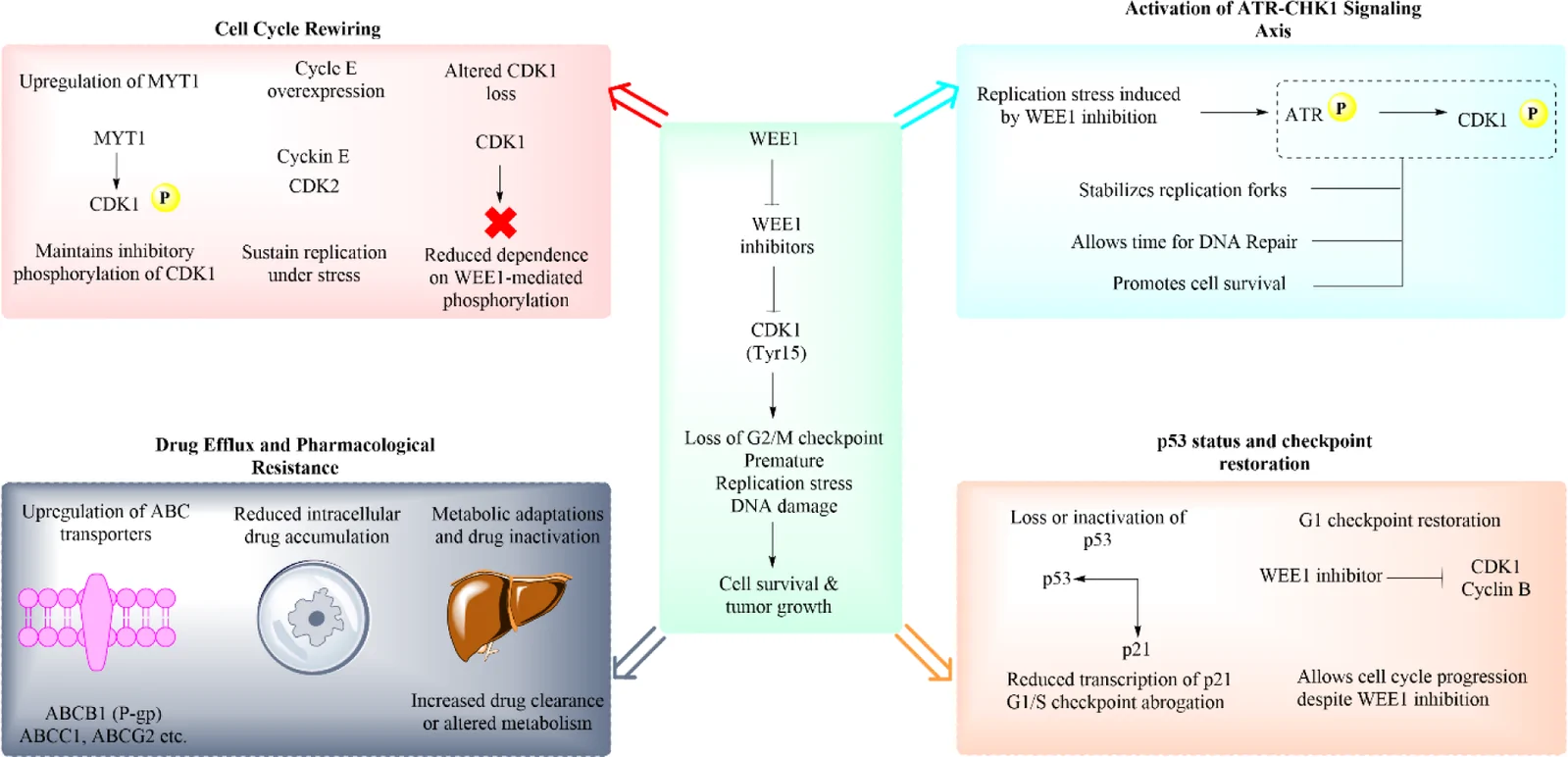
Potential resistance mechanisms limiting the efficacy of WEE1 inhibitors in cancer cells

Proteomic analyses have also revealed that WEE1 inhibitors such as AZD1775 exhibit off-target activity against kinases like PLK1, suggesting that adaptive kinase network rewiring and target redundancy may further modulate cellular responses and therapeutic efficacy (Wright et al., 2017[75]). Collectively, these findings indicate that cancer cells maintain cell-cycle progression and genome integrity through redundant checkpoint kinases and compensatory signaling networks, thereby limiting the effectiveness of WEE1-targeted therapies and highlighting the importance of rational combination strategies. Figure 16(Fig. 16) displays the potential resistance mechanism to WEE1 inhibitors.

## 6. Key Pharmacophoric Features of Most Potent WEE1 Inhibitors

Among all the compiled studies, three compounds named 11, 8, and 13 show the most potent WEE1 inhibition and provide important SAR insights. All three compounds share a heteroaromatic core that is important for hinge-region binding within the WEE1 kinase domain. Compound 11 exhibits an optimal balance of structural features, which enhances both binding affinity and pharmacokinetic performance, resulting in superior overall activity. In contrast, compound 8 achieves good enzymatic potency due to its compact and rigid structure, with increased heteroatom density, which strengthens hydrogen bonding interactions. Meanwhile, compound 13 benefits from an extended bicyclic aromatic system and strategic phenyl substitutions that enhance π-π stacking and hydrophobic interactions within the ATP-binding pocket, leading to a balanced profile of enzymatic and cellular activity. Overall, these findings highlight that while strong hinge binding is critical for potency, the fine-tuning of hydrophobic and polar substituents is essential for achieving optimal biological activity and drug-like characteristics. Table 2(Tab. 2) displays the various features of the top three WEE1 inhibitors.

## 7. Conclusion and Future Prospects

Recent studies on WEE1 inhibitors clearly display that WEE1 is a promising and rational target for cancer therapy, mainly in tumors with TP53 mutations and high replication stress. Various drugs are under clinical trials, but adavosertib (AZD1775) is the most advanced and widely studied compound. It has progressed through multiple Phase I/II trials with encouraging results in ovarian and uterine cancers. From the compounds discussed in this manuscript, two compounds, **compound 8** and **compound 11,** are the most potent. **Compound 8** displayed extremely strong WEE1 inhibitory activity in the nanomolar range, while **compound 11** showed a *K*_i_ value below 1.0 nM along with excellent oral bioavailability. Compound 11 also displayed significant *in vivo* tumor growth inhibition. Based on the SAR studies, it has been found that electron-withdrawing groups play an important role in enhancing binding affinity within the ATP-binding pocket of WEE1 by improving potency. Collectively, these findings suggest that further optimization in structural features along with biomarker-driven clinical strategies may lead to the development of more effective and selective WEE1-targeted inhibitors.

Despite promising clinical progress of various WEE1 inhibitors, some challenges, such as the development of resistance, haematological toxicity, and limited single-agent efficacy, remain unresolved. Continuous efforts are being made by many researchers, but future research should be focused on combination strategies using WEE1 inhibitors with ATR, CHK1, and PARP inhibitors to exploit mechanism-based targeting. Hematological toxicity represents a major clinical limitation associated with WEE1 inhibitors, particularly in adavosertib (AZD1775) clinical trials (Takebe et al., 2021[62]). Mechanistically, WEE1 inhibition abrogates the G2/M checkpoint, forcing cells with unrepaired DNA damage into premature mitosis, which not only affects tumor cells but also rapidly proliferating normal hematopoietic progenitors in the bone marrow. This results in dose-limiting myelosuppression, characterized by anemia, neutropenia, leukopenia, lymphopenia, and thrombocytopenia (Guler et al., 2023[20]). Clinical evidence indicates that hematologic adverse events are among the most frequent toxicities, with anemia (69 %), lymphopenia (71 %), and leukopenia (50 %) commonly observed, and severe grade 3/4 toxicities including neutropenia and leukopenia reported in a significant proportion of patients. Notably, grade 4 toxicities associated with WEE1 inhibition are almost exclusively haematological, underscoring the vulnerability of bone marrow cells to checkpoint abrogation (Takebe et al., 2021[62]). Additionally, off-target kinase inhibition (e.g., PLK1) has been suggested to contribute to myelosuppression, although even highly selective WEE1 inhibitors still demonstrate hematologic toxicity, indicating that this effect is at least partly mechanism-based. Combination therapies, particularly with DNA-damaging agents such as platinum compounds, further exacerbate haematological adverse effects due to synergistic induction of replication stress and DNA damage in normal cells (Guler et al., 2023[20]). Recent articles emphasize that hematologic toxicity remains a key barrier to dose optimization and long-term therapeutic use. Consequently, strategies such as intermittent dosing schedules, biomarker-guided patient selection, and the development of more selective inhibitors are being explored to mitigate these adverse effects while maintaining antitumor efficacy (Zhang et al., 2024[80]). Recent phase Ib studies further confirm that severe (grade ≥ 3) hematologic toxicities such as anemia and neutropenia remain frequent, occurring in approximately 14-15 % of patients and often leading to dose interruptions or reductions (Falchook et al., 2023[13]). In pediatric and combination settings, profound hematologic toxicity has also been observed, including prolonged grade 4 neutropenia and transfusion-dependent thrombocytopenia, highlighting the synergistic toxicity arising from enhanced replication stress (Gatz et al., 2024[16]). Collectively, these findings highlight that haematological toxicity is an intrinsic, mechanism-driven limitation of WEE1 inhibition, posing significant challenges for dose optimization and long-term therapeutic application. Therefore, future strategies focusing on selective targeting, optimized dosing regimens, and biomarker-guided patient stratification will be essential to improve the therapeutic window while minimizing hematopoietic toxicity. Researchers should also focus on development of next-generation WEE1 inhibitors with improved selectivity and PK profiles. Additionally, structural optimization guided by SAR displayed that incorporation of strategically positioned electron-withdrawing groups can further enhance potency and metabolic stability of the compounds. Emerging approaches such as molecular glue and PROTAC-mediated WEE1 degradation also provide durable target suppression beyond catalytic inhibition.

## Notes

Keshav Raj Paudel, Rajwinder Kaur (Chitkara College of Pharmacy, Chitkara University, Rajpura-140401, Punjab, India; E-mail: rajwinder.kaur@chitkara.edu.in) and Rohit Bhatia (Chitkara College of Pharmacy, Chitkara University, Rajpura-140401, Punjab, India; E-mail: bhatiarohit5678@gmail.com) contributed equally as corresponding author.

## Declaration

### Author contributions

**Conceptualization:** A.K., K.R.P., R.K., R.B.; **Validation:** K.R.P., R.K., R.B.; **Investigation:** A.K.; **Resources:** R.K., R.B.; **Data Curation:** A.K.; **Writing - Original draft preparation:** A.K.; **Writing - Review and Editing and Visualization:** K.R.P., R.K., R.B.; **Supervision:** K.R.P., R.K., R.B. All the authors read and approved the final version of the manuscript.

### Conflict of interest

The authors declare no conflict of interest.

### Artificial Intelligence (AI) - assisted technology

The authors declare that no artificial intelligence (AI) tools or technologies were used in the preparation, writing, or analysis of this manuscript.

## Figures and Tables

**Table 1:**
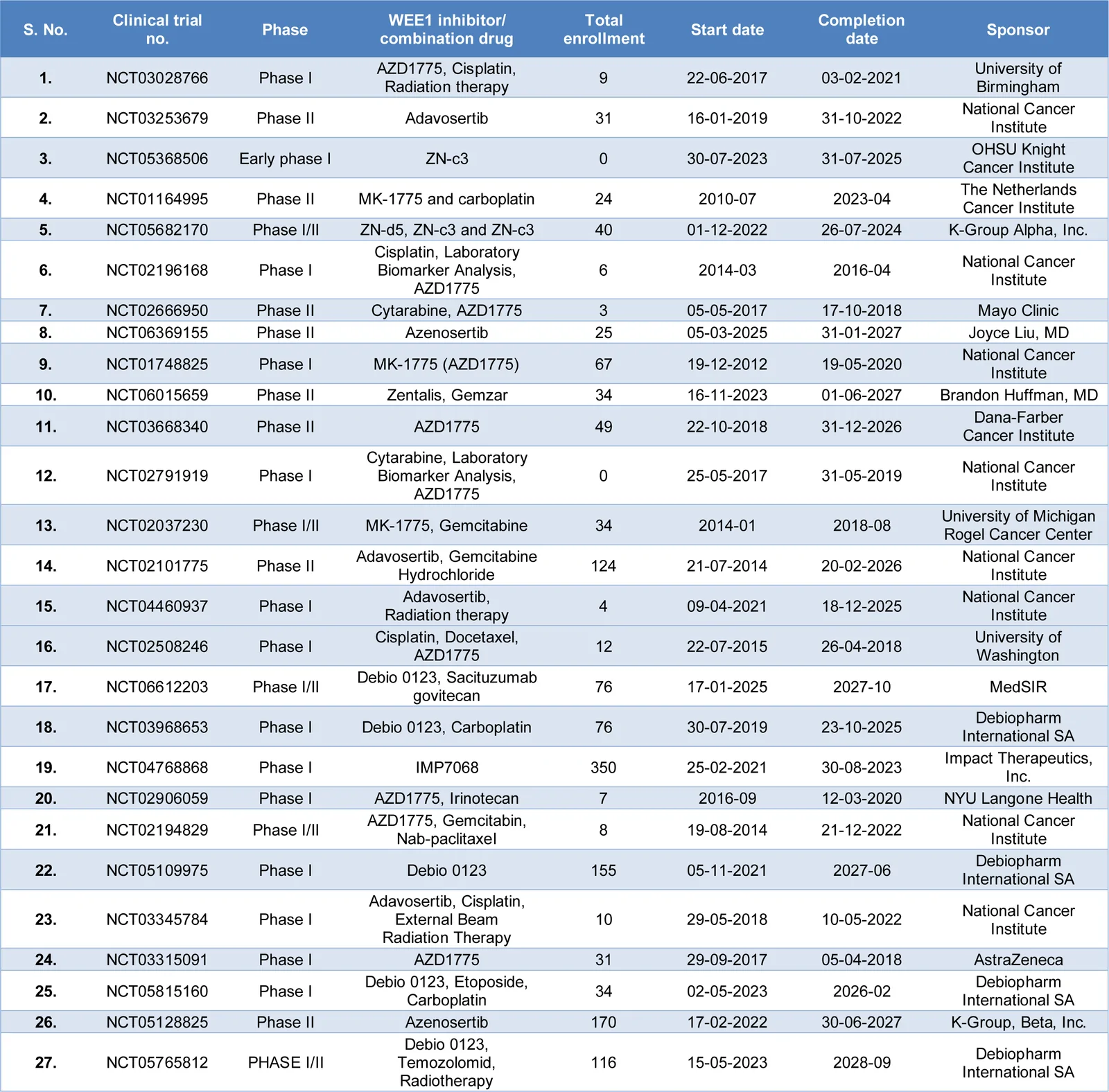
WEE1 inhibitors under clinical trials

**Table 2:**
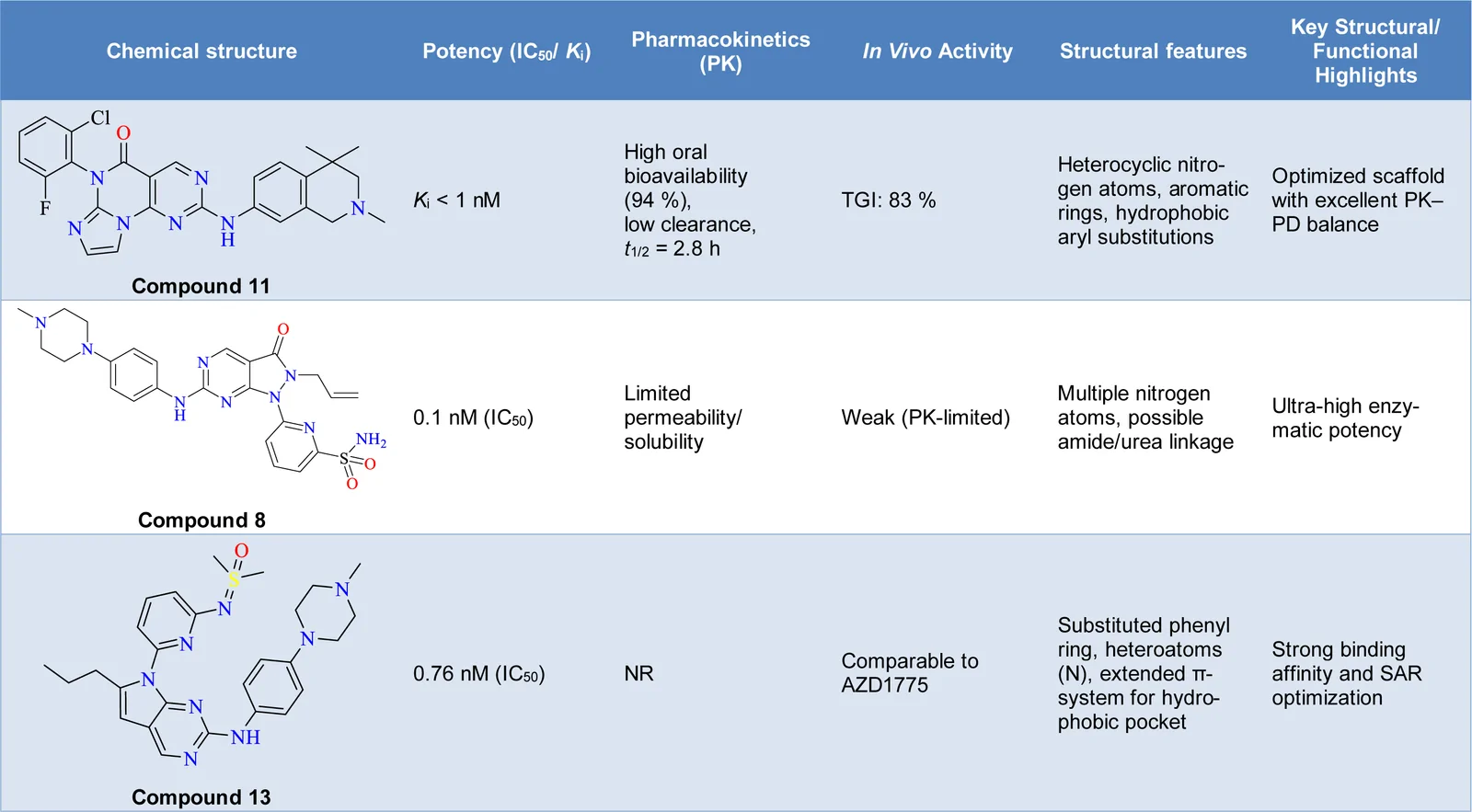
Top three potent WEE1 inhibitors

**Figure 1:**
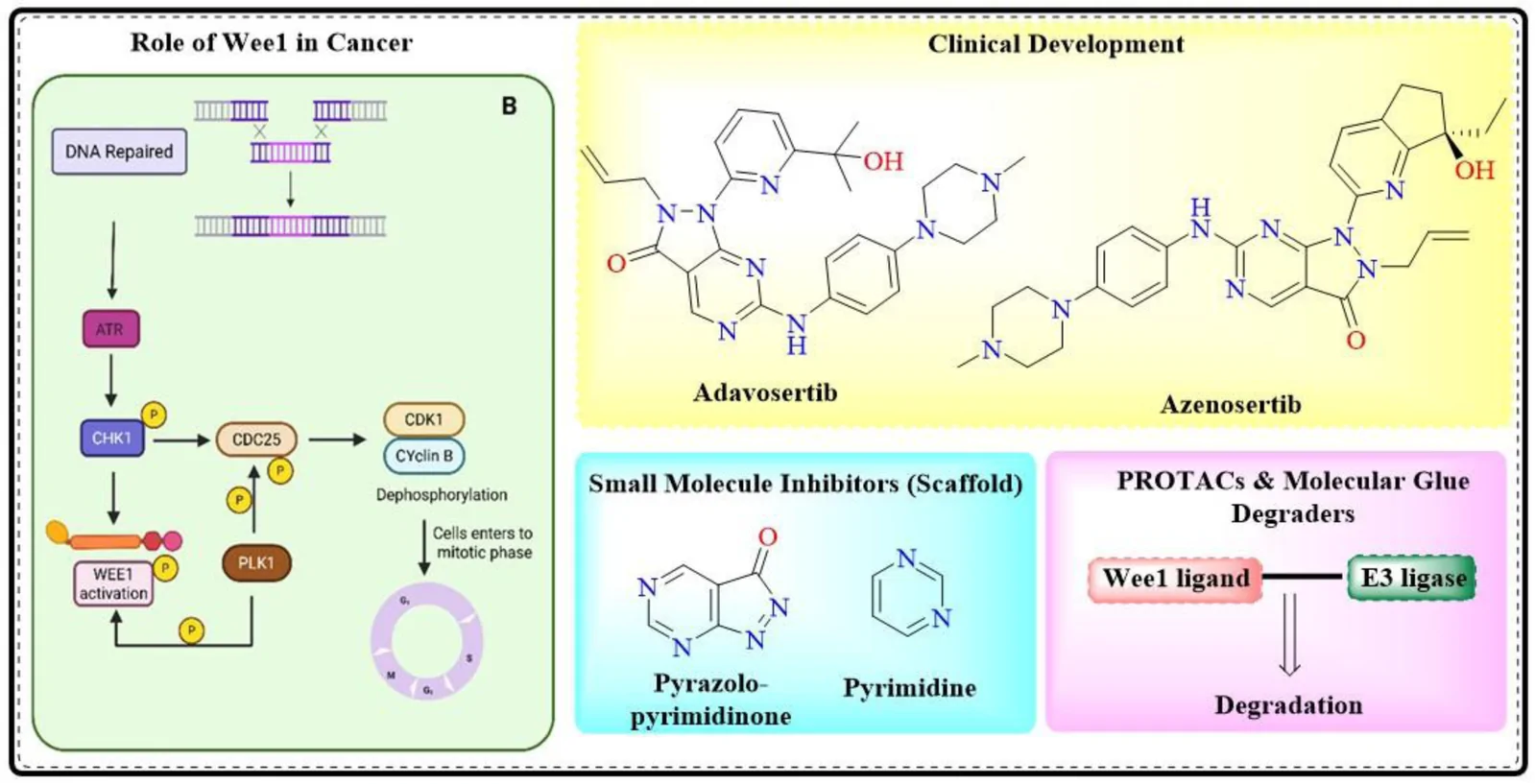
Graphical abstract
